# Three Gd-based magnetic refrigerant materials with high magnetic entropy: From di-nuclearity to hexa-nuclearity to octa-nuclearity

**DOI:** 10.3389/fchem.2022.963203

**Published:** 2022-09-29

**Authors:** Minmin Wang, Chengyuan Sun, Yujia Gao, Hong Xue, Ling Huang, Yutian Xie, Jin Wang, Yuanyuan Peng, Yanfeng Tang

**Affiliations:** ^1^ School of Chemistry and Chemical Engineering, Nantong University, Nantong, China; ^2^ Department of Chemistry, Southern University of Science and Technology, Shenzhen, China

**Keywords:** magnetocaloric effect, polynuclear, lanthanide, Schiff-based ligand, magnetic entropy

## Abstract

Magnetocaloric effect (MCE) is one of the most promising features of molecular-based magnetic materials. We reported three Gd-based magnetic refrigerant materials, namely, Gd_2_(L)(NO_3_)(H_2_O)‧CH_3_CN‧H_2_O (**1**, H_2_L = (*Z*)-*N*-[(1*E*)-(2-hydroxy-3-methphenyl)methylidene]pyrazine-2-carbohydrazonic acid), {Gd_6_(L)_6_(CO_3_)_2_(CH_3_OH)_2_(H_2_O)_3_Cl}Cl‧4CH_3_CN (**2**), and Gd_8_(L)_8_(CO_3_)_4_(H_2_O)_8_‧2H_2_O (3). Complex **1** contains two Gd^III^ ions linked by two *η* ^2^:*η* ^1^:*η* ^1^:*η* ^1^:*μ*_2_-L^2-^ ligands, which are seven-coordinated in a capped trigonal prism, and complex **2** possesses six Gd^III^ ions, contributing to a triangular prism configuration. For complex **3**, eight Gd^III^ ions form a distorted cube arrangement. Moreover, the large values of magnetic entropy in the three complexes prove to be excellent candidates as cryogenic magnetic coolants.

## Introduction

Ln-based complexes play a critical role in molecular-based materials not only due to the charming geometrical structures but also because of the extensive applications such as luminescence, catalysis, especially for magnetic materials including magnetocaloric effect (MCE) (Wu D et al., 2020; Shang et al., 2021; Wei et al., 2021), and single-molecule magnets (SMMs) (Liu et al., 2014; Liu et al., 2016; Zhang and Cheng, 2016; Reis, 2020). As a member of the Ln elements, the Gd ion is a perfect candidate in the synthesis of molecular-based magnetic refrigeration materials because of the large magnetothermal effects (Evangelisti et al., 2011; Chen et al., 2013; Chen et al., 2014; Wang et al., 2020a; Li et al., 2021; Lin et al., 2021; Wu T et al., 2021; Zhou et al., 2021). Some of the reported magnetic materials even possess a large cryogenic MCE, which is comparable to that of the commercial coolant {Gd_3_Ga_5_O_12_} (Pecharsky and Gschneidner, 1997; Zhang S. et al., 2015; Zhang S.,-W. et al., 2015).

It is worth mentioning that in the pure 4f system, improving magnetic density is the ideal method to gain MCE performance (Zhang et al., 2016; Reis, 2020). Therefore, organic ligands play an important role in the building units of the complexes. In previous studies, various organic ligands (e. g. Schiff-based ligands (Aronica et al., 2006; Boulon et al., 2013; Mannini et al., 2014; Burgess et al., 2015; Nava et al., 2015; Wang et al., 2015; Lakma et al., 2019; Li et al., 2019; Wang et al., 2020b; Wang J. et al., 2021; Wang M. et al., 2021), carboxylates (Milios et al., 2007; Dermitzaki et al., 2015; Yin et al., 2015; Botezat et al., 2017; Feltham et al., 2017; Li et al., 2019; Zheng et al., 2020; Han et al., 2021; Zhou et al., 2021), diketones (Zhu et al., 2014; Yao et al., 2018; Wang et al., 2019a; Wang et al., 2019b; Shi et al., 2021), and diamines (Neves et al., 1992; Zhang et al., 2013; Cornia et al., 2014; Oyarzabal et al., 2014; Feltham et al., 2015; Luan et al., 2015; Lu et al., 2019) etc.) have been successfully utilized in the synthesis of MCE materials. Among them, Schiff-based ligands comprise rich O and N sites, which are widely used in the synthesis of many Ln complexes because of the simple synthesis and structural diversity.

In this work, three Gd-based magnetic refrigerant materials based on Schiff-based ligands (*Z*)-*N*-[(1*E*)-(2-hydroxy-3-methphenyl) methylidene]pyrazine-2-carbohydrazonic acid (H_2_L) were synthesized, namely, Gd_2_(L) (NO_3_) (H_2_O)‧CH_3_CN‧H_2_O (**1**), {Gd_6_(L)_6_(CO_3_)_2_(CH_3_OH)_2_(H_2_O)_3_Cl}Cl‧4CH_3_CN (**2**), and Gd_8_(L)_8_(CO_3_)_4_(H_2_O)_8_‧2H_2_O (**3**). Magnetic studies indicate that all complexes exhibit antiferromagnetic interactions between the spin centers and display large magnetic entropies.

## Materials and methods

### Materials

All reactions and manipulations were performed in the ambient atmosphere. The Schiff-based H_2_L ligand was prepared by condensation with *o*-vanillin and hydrazine-2-carbohydrazide in methanol according to the literature (Chandrasekhar et al., 2013; Chen et al., 2016). Metal salts and other reagents were commercially available and used without further purification.

### Synthesis

Synthesis of Gd_2_(L)_2_(NO_3_)_2_(H_2_O)_2_‧CH_3_CN‧H_2_O (**1**): a mixture of H_2_L (0.1 mmol, 27.2 mg) and Gd(NO_3_)_3_·6H_2_O (0.1 mmol, 45.7 mg) was dissolved in CH_3_CN (5 ml) and CH_3_OH (2.5 ml). After stirring for 5 min, pyridine (0.04 ml) was added and stirred for another 10 min. The solution was filtered and left to slowly evaporate. Well-shaped orange crystals were obtained after 1 week. Yield: 20 mg, 36% based on Gd. Elemental analysis (EA) calc. (%) for Gd_2_C_30_H_30_N_12_O_16_, C: 31.91, H: 2.68, N: 14.89; found (%), C: 32.03, H: 2.61, N: 14.93.

{Gd_6_(L)_6_(CO_3_)_2_(CH_3_OH)_2_(H_2_O)_3_Cl}Cl‧4CH_3_CN (2): a mixture of H_2_L (0.2 mmol, 54.4 mg) and GdCl_3_.6H_2_O (0.2 mmol, 74.3 mg) was dissolved in CH_3_CN (10 ml) and CH_3_OH (5 ml). After stirring for 5 min, NaHCO_3_ (0.2 mmol, 33.6 mg) was added and stirred for another 3 h. Well-shaped orange crystals were obtained after 1 week. Yield: 32 mg, 32% based on Gd. Elemental analysis (EA) calc. (%) for Gd_6_C_90_H_92_N_28_O_29_Cl_2_, C: 35.51, H: 3.05, N: 12.88; found (%), C: 35.72, H: 2.99, N: 12.92.

Gd_8_(L)_8_(CO_3_)_4_(H_2_O)_8_‧2H_2_O (3): a mixture of H_2_L (0.2 mmol, 13.6 mg) and GdCl_3_.6H_2_O (0.2 mmol, 18.6 mg) was dissolved in CH_3_CN (5 ml) and CH_3_OH (2.5 ml). After stirring for 5 min, NaCO_3_ (0.2 mmol, 10.6 mg) was added and stirred for another 2 h. Well-shaped orange crystals were obtained after 1 week. Yield: 28 mg, 29% based on Gd. Elemental analysis (EA) calc. (%) for Gd_8_C_108_H_100_N_32_O_46_, C: 33.78, H: 2.62, N: 11.67; found (%), C: 33.83, H: 2.51, N: 11.84.

### Physical measurements

The C, H, and N elemental analyses were performed using an Elementar Vario-EL CHNS elemental analyzer. The Fourier transform-infrared (FT-IR) spectra were carried out from KBr pellets in the range 4,000–400 cm^−1^ using an EQUINOX 55 spectrometer. Powder X-ray diffraction (PXRD) patterns were performed using the Bruker D8 Advance diffractometer (Cu–*K*α, *λ* = 1.54056 Å). Magnetic susceptibility measurements were measured with a Quantum Design MPMS-XL7 SQUID. Polycrystalline samples were embedded in vaseline to prevent torquing. Data were corrected for the diamagnetic contribution calculated from Pascal constants.

### Crystallographic study

Suitable single crystals for **1–3** were selected for single-crystal X-ray diffraction analysis. Data were collected using a Rigaku Oxford diffractometer with a Mo–K*α* radiation (*λ* = 0.71073 Å) at 120 K. The structures were solved by direct methods and refined by least-squares on *F* ^2^ utilizing the SHELXTL program suite and Olex2 (Dolomanov et al., 2009; Sheldrick, 2015a,b). The hydrogen atoms were set in calculated positions and refined as riding atoms with common fixed isotropic thermal parameters. EA was used to detect the content of C, H, and N atoms. Detailed information about the crystal data and structure refinements is summarized in Table 1. Selected bond lengths and angles of complexes **1–3** are listed in Supplementary Table S1–S3.

**TABLE 1:** Crystallographic data and structural refinement parameters for complexes **1–3**.

Complex	1	2	3
Formula	Gd_2_(L)_2_(NO_3_)_2_(H_2_O)_2_‧CH_3_CN‧H_2_O	{Gd_6_(L)_6_(CO_3_)_2_(CH_3_OH)_2_(H_2_O)_3_Cl}Cl‧4CH_3_CN	Gd_8_(L)_8_(CO_3_)_4_(H_2_O)_8_‧2H_2_O
*M*_r_ [g·mol^−1^]	1127.17	3044.31	3840.19
*T* [K]	120 (2)	120 (2)	120 (2)
Crystal system	Triclinic	Triclinic	Triclinic
Space group	*P*-1	*P*-1	*P*-1
*a* [Å]	8.8137 (8)	13.5408 (9)	17.84766 (16)
*b* [Å]	9.4123 (9)	18.8679 (15)	18.2321 (2)
*c* [Å]	13.3026 (12)	23.2844 (16)	28.0616 (3)
*α* [°]	95.912 (3)	90.424 (3)	73.0978 (10)
*β* [°]	109.101 (3)	92.888 (2)	77.7530 (8)
*γ* [°]	107.900 (3)	106.296 (2)	61.2890 (11)
*V* [Å^3^]	966.55 (16)	5701.2 (7)	7634.03 (16)
*Z*	1	2	2
*ρ*_calcd_ [g·cm^−3^]	1.936	1.773	1.671
*μ* [mm^−1^]	3.487	3.569	3.506
*F* (000)	550.7	2956.0	3704.0
Refl.collected/unique	8376/3895	70871/25093	124216/39110
GOF on *F* ^2^	1.0385	1.030	1.033
*R*_1_/*wR*_2_ [*I* > 2*σ*(*I*), squeeze] *^a^*	0.0379/0.0963	0.0393/0.0943	0.0378/0.0887
*R*_1_/*wR*_2_ (all data, squeeze)	0.0424/0.1001	0.0454/0.0995	0.0512/0.0946
CCDC No.	2174539	2174540	2174541

a *R*_1_ = *∑*||*F*_o_| − |*F*_c_||/*∑*|*F*_o_|. *wR*_2_ = [*∑w* (*F*_o_^2^–*F*_c_^2^)^2^/*∑w* (*F*_o_^2^)^2^]^1/2^.

## Results and discussion

### Description of the structures of **1–3**

**SCHEME 1:**
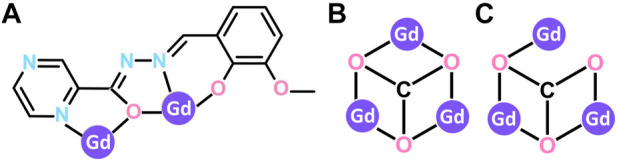
Coordination modes of L^2-^ ligand **(A)** and CO_3_^2-^ **(B,C)**.

Complexes **1–3** are synthesized by the evolution method with H_2_L and gadolinium salt in the solution of CH_3_CN/CH_3_OH (*V*_1_:*V*_2_ = 2:1) under the existence of alkali. The alkali is added to be conducive to protonate the ligand H_2_L, which is beneficial to incorporate Gd^III^ ions. The H_2_L ligand in all complexes is completely dehydrogenated adopting the *μ*_2_:*η* ^2^:*η* ^1^:*η* ^1^:*η* ^1^-mode (Scheme 1A), which is similar to the reported literature (Chandrasekhar et al., 2013; Chen et al., 2016; Zhang et al., 2017; Zhang  et al., 2016; Jiang et al., 2016).

**FIGURE 1:**
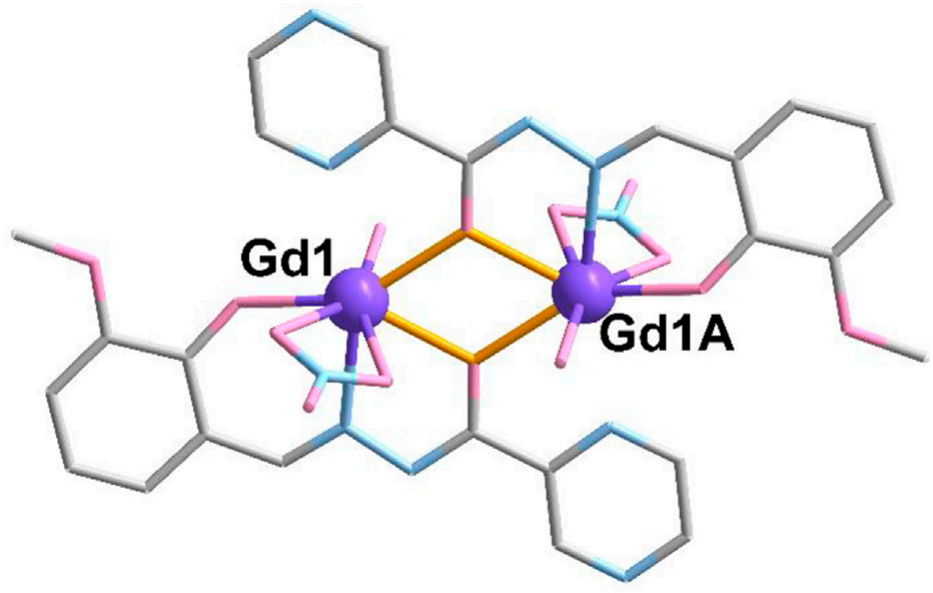
Crystal structure of complex **1**. The hydrogen atoms are omitted for clarity. Color codes: Gd, purple; O, pink; N, blue; and C, grey. Symmetric code: A, 1-x, 1-year, and 1-z.

Complex **1** is crystalized in the triclinic *P*-1 space group. As shown in Figure 1, the crystallography independent unit of **1** contains half of the molecule, including one Gd^III^ ion, one L_2_^-^ ligand, one NO_3_^−^ anion, and half of CH_3_CN and H_2_O molecules. The metallic Gd^III^ ions (Gd1 and Gd1A) are surrounded by two L_2_^-^ ligands using the aforementioned mode, two NO_3_^−^ anions and two H_2_O molecules located above and below the plane, respectively. The average bond lengths of Gd-O and Gd-N are 2.379 (5) Å and 2.460 (5) Å (Supplementary Table S1), respectively, which are in accordance with those of the reported Gd-based complexes (Chen et al., 1995; Zhao et al., 2017; Mayans and Escuer, 2021; Ren et al., 2021). In complex **1**, the Gd ion is seven-coordinated to form a capped trigonal prism, which is confirmed by CShM calculations (Alvarez et al., 2005; Casanova et al., 2005) (Supplementary Figure S1, Supplementary Table S4).

**FIGURE 2:**
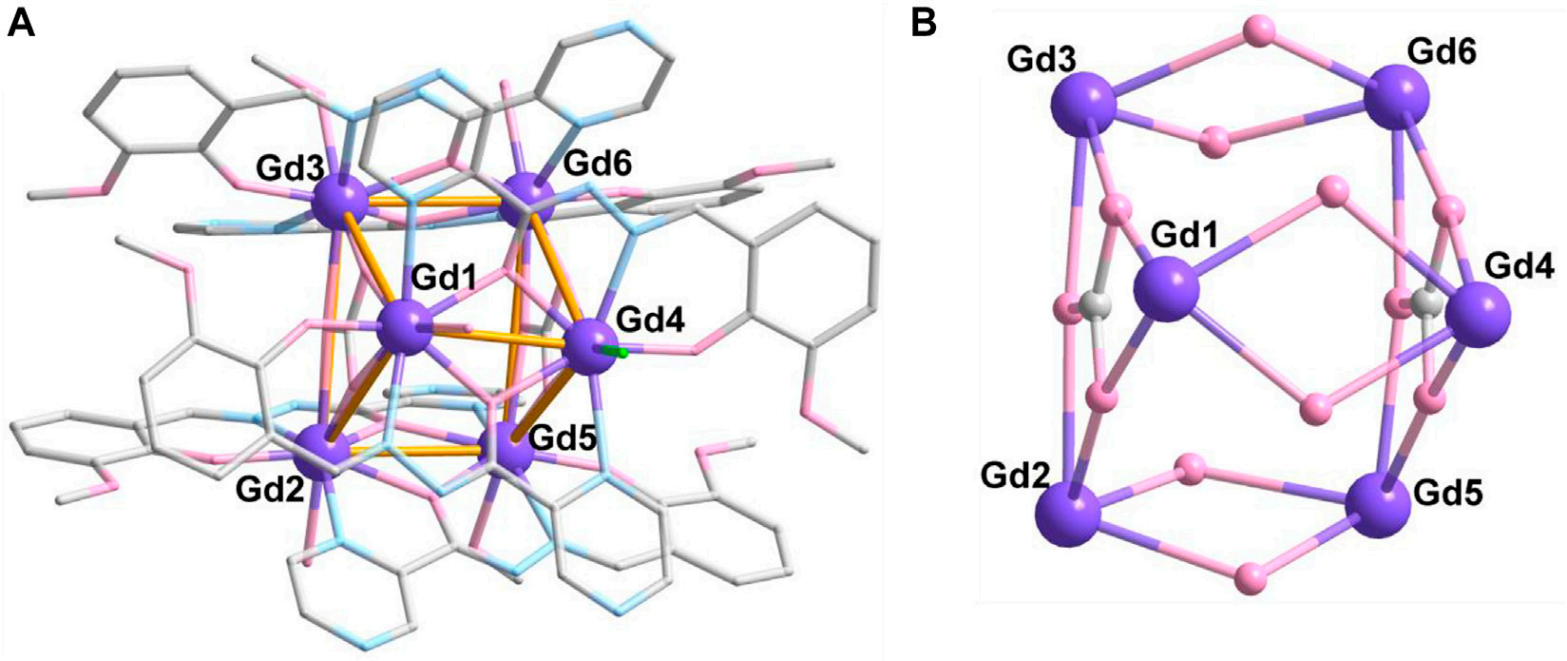
Crystal structure **(A)** and metallic core **(B)** of complex **2**. The hydrogen atoms are omitted for clarity. Color codes: Gd, purple; O, pink; N, blue; and C, gray.

Complex **2** crystalizes in the same space group as complex **1**, and the asymmetric unit comprises the whole molecule with six crystallographically independent Gd^III^ ions (Figure 2A). The six Gd ions are held together to form a {Gd_6_} triangular prism metallic skeleton (Figure 2B). Therein, three Gd ions in the plane (Gd1, Gd2, and Gd3 or Gd4, Gd5, and Gd6) contribute a triangular configuration, which are bridged by one CO_3_^2-^ anion in *μ*_3_-*η* ^2^:*η* ^2^:*η* ^2^-mode (Scheme 1B). The two triangular metallic skeletons are then linked together by six *μ*_2_-O bridges from ligands.

All Gd ions are eight coordinated, showing two kinds of coordination geometry confirmed by CShM calculations (Alvarez et al., 2005; Casanova et al., 2005) (Supplementary Table S5). The Gd1, Gd2, Gd3, Gd5, and Gd6 ions are in {O_6_N_2_} environment with six O atoms and two N atoms from two chelated L^2-^ ligands, one CO_3_^2-^ anion and one CH_3_OH/H_2_O molecule, which display a biaugmented trigonal prism configuration (Supplementary Figure S2). The average Gd-O and Gd-N distances are 2.352 (4) Å and 2.475 (4) Å, respectively (Supplementary Table S2), which are consistent with those reported Gd-based complexes (Chen et al., 1995; Zhao et al., 2017; Mayans and Escuer, 2021; Ren et al., 2021). However, Gd4 has triangular dodecahedron coordination geometry and is located in an {O_5_N_2_Cl} environment with five O and two N atoms from two chelated L^2-^ ligands and one Cl^−^ anion. The bond length of Gd4-Cl1 is 2.746 (1) Å, which is longer than that of Gd-O and Gd-N.

**FIGURE 3:**
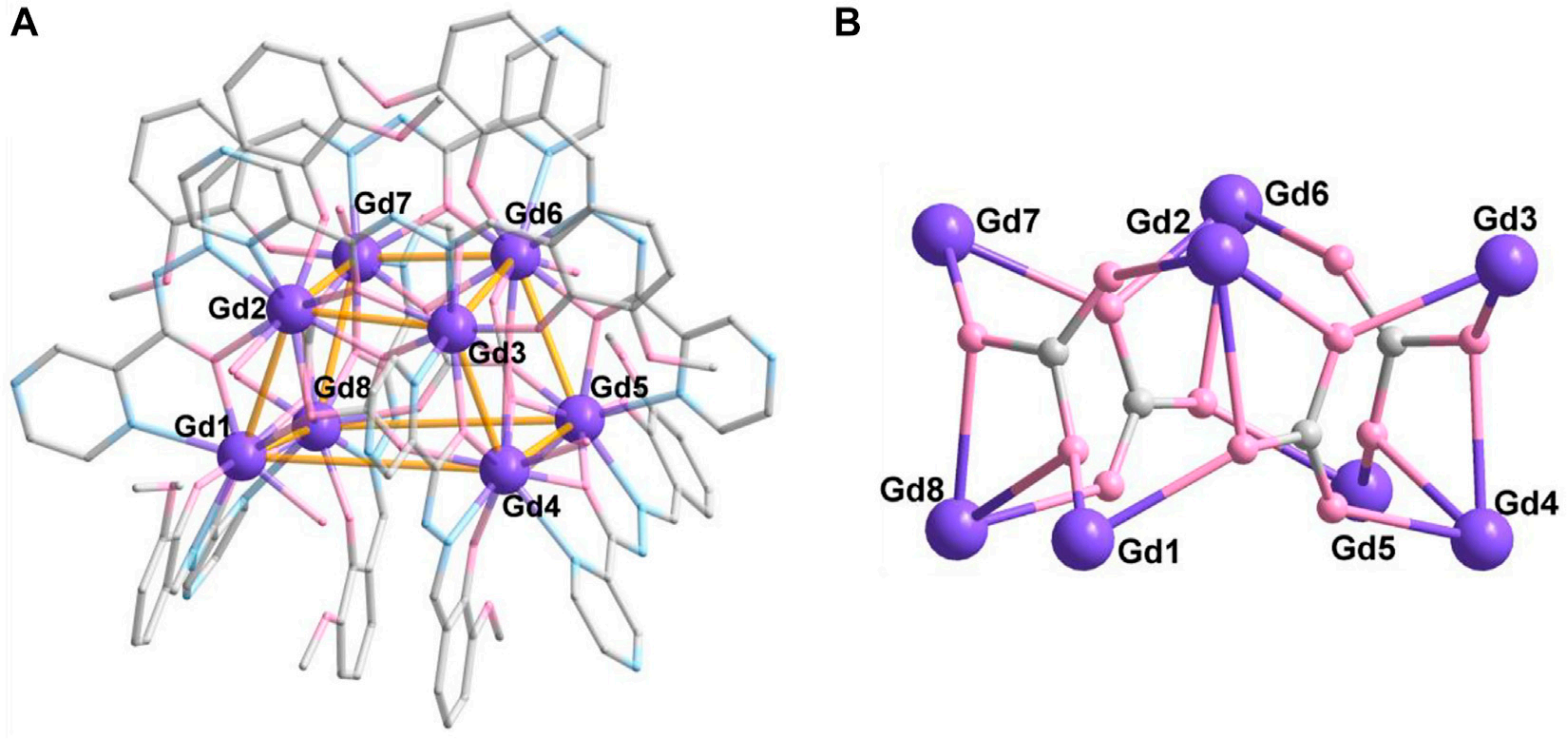
Crystal structure **(A)** and metallic core **(B)** of **3**. The hydrogen atoms are omitted for clarity. Color codes: Gd, purple; O, pink; N, blue; and C, gray.

For complex **3**, the synthetic method is the same as complex **2**; except NaHCO_3_ was used in place of Na_2_CO_3_. Surprisingly, complex **3** possesses an octa-nuclearity structure, which crystalizes in the triclinic *P*-1 space group. The asymmetric unit consists of a completed molecule, and there are eight crystallographically independent Gd atoms in the molecular structure (Figure 3A). As shown in Figure 3B, the eight Gd^III^ ions contribute to a cubic trapezoid metallic core. Gd1, Gd4, Gd5, and Gd8 ions lie in the four vertices of the plane below the cubic trapezoid, while Gd2, Gd3, Gd6, and Gd7 ions situate in the upper plane. The metallic core is held together by four CO_3_^2-^ anions in *μ*_3_-*η* ^2^:*η* ^2^:*η* ^1^-mode (Scheme 1C). The periphery of the metal core is ligated by eight L^2-^ ligands, eight H_2_O molecules, and two lattice H_2_O molecules.

There are two coordination numbers of Gd^III^ ions in complex **3** (Supplementary Figure S3). Gd1, Gd3, Gd5, and Gd7 are eight-coordinated ions in {O_6_N_2_} donor set from two L^2-^ ligands, two CO_3_^2-^ anions, and one H_2_O molecule, while Gd2, Gd4, Gd6, and Gd8 ions are nine-coordinated in the {O_7_N_2_} donor set. The difference between the two kinds of Gd ions is the diverse coordination modes of the CO_3_^2-^ anion. There is only one coordination bond of O atom in CO_3_^2-^ anion, which is adopted in Gd1, Gd3, Gd5, and Gd7 ions. For Gd2, Gd4, Gd6, and Gd8 ions, the bonding mode of the CO_3_^2-^ anion is adopted in the bidentate mode. The eight metal ions exhibit three coordination geometries: biaugmented trigonal prism (Gd1), triangular dodecahedron (Gd3, Gd5, and Gd7), and muffin (Gd2, Gd4, Gd6, and Gd8) (Supplementary Tables S5,6). The average Gd-O distance is 2.361 (4) Å, which is shorter than that of Gd-N (2.564 (4) Å) lengths. The O/N-Gd-O/N angles are in the range of 60.99°–154.86°, which are in the normal range (Chen et al., 1995; Zhao et al., 2017; Mayans and Escuer, 2021; Ren et al., 2021).

It is worth mentioning that the use of different alkalis can affect the number of formed metal nuclearity. For the organic weak alkali triethylamine, which is used in complex **1**, it only facilitates protonation of the ligand H_2_L but is not involved in the final formation of complex **1**. However, for complexes **2** and **3**, the inorganic alkalis not only deprotonate the ligand but also participate in the construction of the molecules. Compared to NaHCO_3_ in complex **2**, the alkalinity of Na_2_CO_3_ is relatively strong. Moreover, mainly due to the degree of hydrolysis of carbonates being higher, there are more carbonate triangle skeletons in complex **3**, making it easier to coordinate with Gd ions, thus forming an octa-nuclearity complex.

### IR spectra and PXRD studies

The FT-IR spectra of complexes **1–3** were acquired (*v* = 4,000–500 cm^−1^), which are shown in Supplementary Figure S4. Powder X-ray diffraction (PXRD) measurements for complexes **1–3** were performed for the crystalline crystals (Supplementary Figure S5), and the experimental patterns are in good agreement with the simulated ones from the crystallographic data. The minor inconsistencies in the intensity and shape of the peaks indicate the phase purity of complexes **1–3**.

### Magnetic studies

**FIGURE 4:**
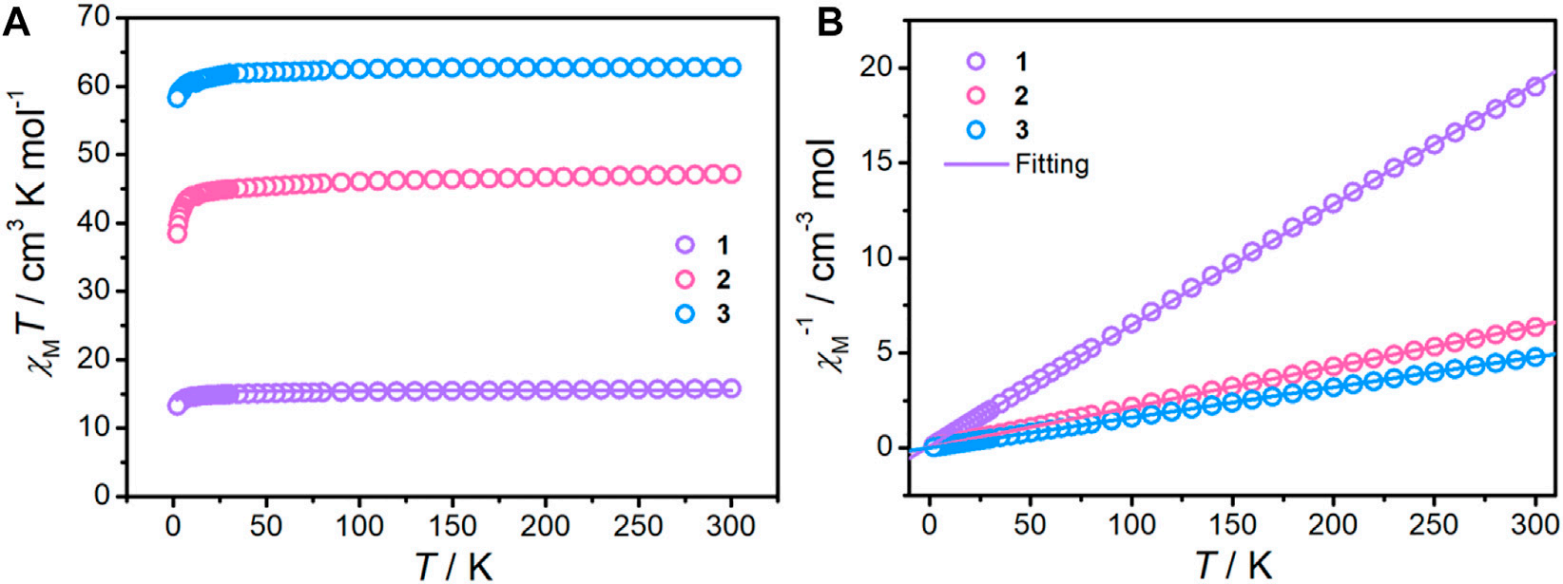
*χ*_M_ *T* products measured under a 1000 Oe DC applied field **(A)** and the plots of 1/*χ*_M_ vs. *T* **(B)** for complexes **1**–**3**. The solid lines represent the best fitting.

The direct current magnetic susceptibilities of complexes **1–3** were studied for polycrystalline samples in the temperature range of 2–300 K at an external magnetic field of 1000 Oe (Figure 4A). At room temperature, the *χ*_M_ *T* values of complexes **1–3** are 15.77, 47.16, and 62.81 cm^3^ K mol^−1^, respectively, which is in good agreement with the expected spin-only values (Gd^III^ ion: 7.875 cm^3^ K mol^−1^, *g* = 2). Upon cooling, the *χ*_M_ *T* values in all cases stay essentially unchanged until approximately 25 K and then followed by an obvious decrease to the minimum values of 13.29, 38.46, and 58.30 cm^3^ K mol^−1^, indicating antiferromagnetic interactions (Kahn et al., 2000). Fitting the curve of *χ*_M_^−1^ vs. *T* with the Curie–Weiss Law (Figure 4B) gives the resulting *C* and *θ* values, which are listed in Supplementary Table S7. The negative *θ* values imply the presence of weak antiferromagnetic interaction within complexes **1–3**.

The field dependence of the magnetization plots for complexes **1–3** was performed in the field range of 1–7 T at 2–8 K (Supplementary Figure S6). Magnetizations in all complexes are increased gradually at the entire field region, reaching saturation values of 13.81, 41.75, and 55.83 *Nμ*_B_ at 7 T and 2 K, respectively, close to the theoretical value (**1**: 14 *Nμ*_B_; **2**: 42 *Nμ*_B_; **3**: 56 *Nμ*_B_). The reduced magnetization plots (*M* vs. *HT* ^−1^) in all complexes are superposable due to the isotropic system (Supplementary Figure S7).

Due to the complicated systems in complexes **2** and **3**, only complex **1** is attempted to analyze the magnetic interactions by using a simplified spin Hamiltonian with the PHI program (Eq. 1):

$${\hat{H}}_{Gd-Gd}=-2J_{Gd-Gd}{\hat{S}}_{Gd1}{\hat{S}}_{Gd2}.$$ (1)

The best-fit parameters are *J* = -0.022 (2) cm^−1^ and *g* = 1.98 (Figure 4A; Supplementary Figure S8). The negative *J* value confirms the antiferromagnetic interactions between the Gd^III^ ions, which is in accordance with the trend of the *χ*_M_ *T* product with cooling and the result of the Curie–Weiss Law.

**FIGURE 5:**
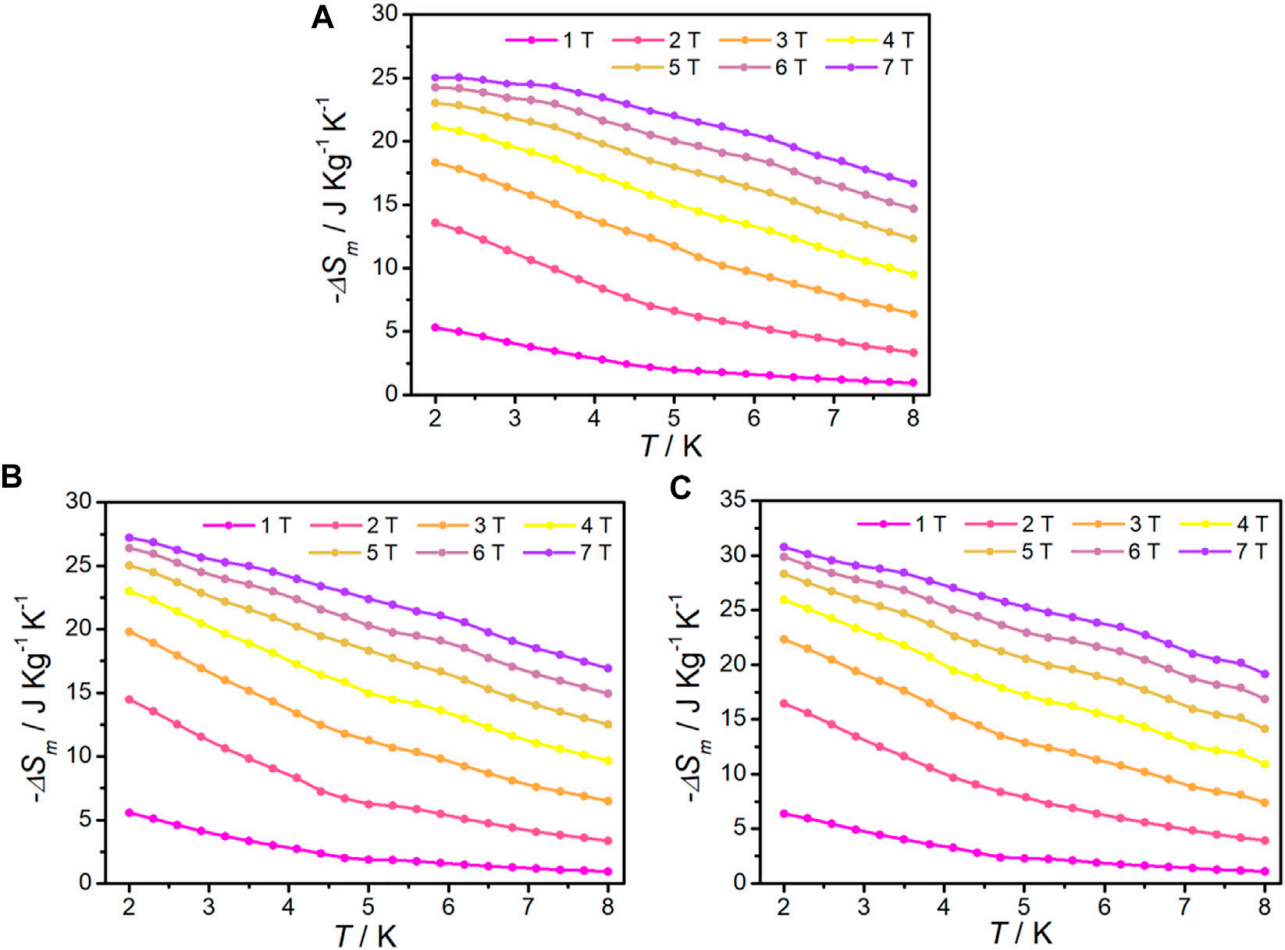
-∆*S*_m_ at various fields and temperatures, calculated from the magnetization data for **1(A)**, **2(B)**, and **3(C)**.

The isothermal magnetization for complexes **1–3** was measured from 2 to 8 K in an applied DC field up to 7 T to calculate the magnetic entropy (-∆*S*_m_) according to the Maxwell equation (Pecharsky and Gschneidner, 1999) (Eq. 2). It can be seen that the curves of -∆*S*_m_ of complexes **1–3** gradually increase with decreasing temperature and increasing of magnetic field without saturation, the maximum -∆*S*_m_ values are 25.05 J kg^−1^ K^−1^, 27.21 J kg^−1^ K^−1^, and 30.79 J kg^−1^ K^−1^ at 2 K, ∆*H* = 7 T, respectively (Figure 5). These values are smaller than the theoretical values of 34.57 J kg^−1^ K^−1^ for **1**, 34.07 J kg^−1^ K^−1^ for **2**, and 36.01 J kg^−1^ K^−1^ for **3**, which are calculated using Eq 3, (*n* = 2, 6, and 8 for **1**, **2**, and **3**, respectively; *S* = 7/2 and the *R* value is 8.314 J mol^−1^ K^−1^), owing to the existence of antiferromagnetic coupling. The maximum -∆*S*_m_ of **1** in di-nuclearity complex is among the highest observed to date for 4f clusters appeared at low temperature (Table 2). Although complexes **2** and **3** do not possess the highest -∆*S*_m_ values, they are still comparable in the same nuclear complexes.

$$\Delta S_{m}\left(T\right)=\int_{0}^{H}{\left[\partial M\left(T,H\right)/\partial T\right]}_{H}dH,$$ (2)

$$\Delta S_{m}\left(T\right)=nR\;\ln\left(2S+1\right).$$ (3)

**TABLE 2:** Summary of -∆*S*_m_ in different ∆*H* at a given temperature for reported di-nuclearity, hexa-nuclearity, octa-nuclearity, and other multinuclear Gd-based complexes.

Complex	-∆*S*_m_ [J kg^−1^ K^−1^]	∆*H* [T], T [K]	Ref
{Gd_2_(OAc)_6_(H_2_O)_4_}·4H_2_O	40.6	7, 1.8	Evangelisti et al. (2011)
Gd_2_(OAc)_2_(Ph_2_acac)_4_ (MeOH)_2_	23.7	7, 2.4	Guo et al. (2012)
Gd_2_ (hfac)_4_ (fpmoq)_2_	17.1	8, 3.0	Wang et al. (2015)
Gd_2_ (hfac)_4_ (btoq)_2_	16.9	8, 2.0	Shen et al. (2015)
Gd_2_ (L_1_) (dbm)_5_	17.69	8, 2.0	Wang et al. (2021a)
Gd_2_ (iba)_6_ (bipy)_2_	29.3	7, 2.0	Zhou et al. (2021)
Gd_2_ (nic)_6_(H_2_O)_4_	27.4	7, 2.0	Zhou et al. (2021)
Gd_2_(L)_2_(CH_3_OH)_2_	24.75	7, 2.0	Shi et al. (2020)
{Gd_2_(L)_2_ (dbm)_2_(H_2_O)_2_}·nCH_3_OH	23.2	7, 2.0	Shi et al. (2021)
Gd_2_ (dnba)_6_ (phen)_2_	16.8	7, 2.0	Zheng et al. (2020)
Gd_2_(Hnsa)_2_ (nsa)_2_ (phen)_2_(H_2_O)_2_	22.2	7, 2.0	Zheng et al. (2020)
**1**	25.05	7, 2.0	This work
{Gd_6_ (bobdz)_2_(HCO_2_)_4_ (μ_3_-OH)_4_ (DMF)_6_(H_2_O)_2_}Cl_2_·4H_2_O	33.5	7,3.0	Adhikary et al. (2014)
Gd_6_(L)_2_ (acac)_6_(OH)_4_(NO_3_)_2_(CH_3_OH)_2_	35.3	7, 2.0	Wang et al. (2020b)
{H_2_ [Gd_6_(OH)_8_(H_2_O)_6_ (p-BDC-F_4_)_6_]}·3 (2,2′-bpy)·6H_2_O	28.27	7, 2.0	Wei et al. (2019)
{H_2_ [Gd_6_(OH)_8_(H_2_O)_6_ (m-BDC-F_4_)_6_]}·3 (4,4′-bpy)·6H_2_O	29.20	7, 2.0	Wei et al. (2019)
**2**	27.21	7, 2.0	This work
{Gd_8_(IN)_14_ (*μ*_3_-OH)_8_ (*μ*_2_-OH)_2_(H_2_O)_8_}·11H_2_O	31.77	7, 2.0	Shi et al. (2020)
{Gd_8_ (*μ*_3_-O)_4_(L)_8_(CH_3_COO)_4_(CO_3_)_2_}·15H_2_O	32.49	7, 2.0	Li et al. (2019)
**3**	30.79	7, 2.0	This work
{Gd_3_ (dbm)_5_(HL)_2_}·4CH_3_OH·3CH_2_Cl_2_	20.60	7, 2.0	Wang et al. (2020c)
{Gd_3_(HL) (H_2_L) (NO_3_)_4_}·C_2_H_5_OH	30.22	7, 2.0	Wang et al. (2019c)
{Gd_4_(H_3_L)_2_(OAc)_3_(F_6_acac)_3_}·4MeOH·2.5H_2_O	21.88	5, 2.0	Liu and Hao, (2022)
{Gd_4_ (acac)_4_ (μ_3_-OH)_2_L_6_}·2CH_3_CN	14.57	7, 3.0	Hou et al. (2020)
{Gd_4_(HL)_4_(CH_3_O)_4_}·3CH_3_OH	30.42	7, 2.0	Li et al. (2020)
{Gd_4_(L)_4_ (m_2_-CH_3_O)_4_}·CH_3_OH	28.50	7, 2.0	Xu et al. (2021)
{Gd_4_ (acac)_4_(L)_6_ (μ_3_-OH)_2_}·CH_3_CN	24.46	7, 2.0	Wang et al. (2020d)

## Conclusion

In conclusion, three clusters **1**-{Gd_2_}, **2**-{Gd_6_}, and **3**-{Gd_8_} based on Schiff ligand H_2_L were synthesized. Complex **1** contains two Gd^III^ ions, and magnetic measurement indicates antiferromagnetic interactions between the metal core, which is also confirmed by PHI fitting. Complexes **2** and **3** are hexa-nuclearity with a biaugmented trigonal prism configuration and octa-nuclearity with a cubic trapezoid structure. Magnetic investigations indicate the antiferromagnetic interactions between Gd^III^ ions are observed in complexes **2** and **3**. Magnetocaloric studies for complexes **1–3** show that the magnetic entropies of complexes **1–3** are smaller than the theoretical values, which is mainly caused by antiferromagnetic coupling. Furthermore, complex **1** exhibits a large magnetic entropy of 25.05 J kg^−1^ K^−1^ at 2.0 K in di-nuclearity magnetic refrigerant materials, while complexes **2** and **3** belong to the normal range in hexa-nuclearity and octa-nuclearity complexes, respectively, demonstrating that they are promising molecular magnetic coolants for low-temperature cooling applications.

## Data Availability

The datasets presented in this study can be found in online repositories. The names of the repository/repositories and accession number(s) can be found in the article/Supplementary Material.
