# Supplementary material for: Three Gd-based magnetic refrigerant materials with high magnetic entropy: From di-nuclearity to hexa-nuclearity to octa-nuclearity
Source: Front Chem. 2022 Sep 29;10:963203. doi: 10.3389/fchem.2022.963203 (PMC9559567; doi:10.3389/fchem.2022.963203)

checkCIF/PLATON report

You have not supplied any structure factors. As a result the full set of tests cannot be run.

THIS REPORT IS FOR GUIDANCE ONLY. IF USED AS PART OF A REVIEW PROCEDURE FOR PUBLICATION, IT SHOULD NOT REPLACE THE EXPERTISE OF AN EXPERIENCED CRYSTALLOGRAPHIC REFEREE.

No syntax errors found. CIF dictionary Interpreting this report

Datablock: Gd6

```
Bond precision:   C-C = 0.0090 A           Wavelength=0.71073

Cell:           a=13.5408(9)       b=18.8679(15)     c=23.2844(16)
                alpha=90.424(3)    beta=92.888(2)    gamma=106.296(2)
Temperature:    120 K
```

	Calculated	Reported
Volume	5701.2(7)	5701.2(7)
Space group	P -1	P -1
Hall group	-P 1	-P 1
Moiety formula	C82 H80 Cl Gd6 N24 O29, 2(C2 H3 N), 2(C2 N), Cl [+ solvent]	C82 H80 Cl Gd6 N24 O29, 2(C2 N), Cl, 2(C2 H3 N)
Sum formula	C90 H86 Cl2 Gd6 N28 O29 [+ solvent]	C90 H92 Cl2 Gd6 N28 O29
Mr	3038.27	3044.31
Dx,g cm-3	1.770	1.773
Z	2	2
Mu (mm-1)	3.569	3.569
F000	2944.0	2956.0
F000'	2943.68	
h,k,lmax	17,24,29	17,24,29
Nref	25302	25093
Tmin,Tmax	0.682,0.725	0.787,0.999
Tmin'	0.669	

```
Correction method= # Reported T Limits: Tmin=0.787 Tmax=0.999
AbsCorr = MULTI-SCAN

Data completeness= 0.992           Theta(max)= 27.153
```

```
R(reflections)= 0.0393( 22239)                              wR2(reflections)=
                                                            0.0995( 25093)
S = 1.030                   Npar= 1415
```

The following ALERTS were generated. Each ALERT has the format
test-name_ALERT_alert-type_alert-level.
Click on the hyperlinks for more details of the test.

Alert level B

```
PLAT213_ALERT_2_B Atom O6             has ADP max/min Ratio .....        4.5 prolat
PLAT220_ALERT_2_B NonSolvent   Resd 1  C   Ueq(max)/Ueq(min) Range       7.0 Ratio
PLAT315_ALERT_2_B Singly Bonded Carbon Detected (H-atoms Missing).       C85 Check
PLAT315_ALERT_2_B Singly Bonded Carbon Detected (H-atoms Missing).       C83 Check
```

Alert level C

```
ABSTY02_ALERT_1_C  An _exptl_absorpt_correction_type has been given without
            a literature citation. This should be contained in the
            _exptl_absorpt_process_details field.
            Absorption correction given as multi-scan
PLAT213_ALERT_2_C Atom C9             has ADP max/min Ratio .....        3.1 oblate
PLAT213_ALERT_2_C Atom C22            has ADP max/min Ratio .....        3.2 prolat
PLAT213_ALERT_2_C Atom C24            has ADP max/min Ratio .....        3.4 prolat
PLAT220_ALERT_2_C NonSolvent   Resd 1  O   Ueq(max)/Ueq(min) Range       5.4 Ratio
PLAT222_ALERT_3_C NonSolvent Resd 1  H   Uiso(max)/Uiso(min) Range       8.0 Ratio
PLAT241_ALERT_2_C High   'MainMol' Ueq as Compared to Neighbors of       C22 Check
PLAT244_ALERT_4_C Low    'Solvent' Ueq as Compared to Neighbors of       C86 Check
PLAT260_ALERT_2_C Large Average Ueq of Residue Including      N27      0.105 Check
PLAT260_ALERT_2_C Large Average Ueq of Residue Including      N84      0.114 Check
PLAT342_ALERT_3_C Low Bond Precision on  C-C Bonds ..............      0.009 Ang.
PLAT414_ALERT_2_C Short Intra D-H..H-X       H3       ..H3A      .      1.99 Ang.
                                                      x,y,z  =      1_555 Check
PLAT420_ALERT_2_C D-H Bond Without Acceptor  N3       --H3       .     Please Check
PLAT420_ALERT_2_C D-H Bond Without Acceptor  N7       --H7       .     Please Check
PLAT420_ALERT_2_C D-H Bond Without Acceptor  N11      --H11      .     Please Check
PLAT420_ALERT_2_C D-H Bond Without Acceptor  N15      --H15      .     Please Check
PLAT420_ALERT_2_C D-H Bond Without Acceptor  N19      --H19      .     Please Check
PLAT420_ALERT_2_C D-H Bond Without Acceptor  N23      --H23      .     Please Check
```

Alert level G

```
FORMU01_ALERT_1_G  There is a discrepancy between the atom counts in the
            _chemical_formula_sum and _chemical_formula_moiety. This is
            usually due to the moiety formula being in the wrong format.
            Atom count from _chemical_formula_sum:   C90 H92 Cl2 Gd6 N28 O29
            Atom count from _chemical_formula_moiety:C90 H86 Cl2 Gd6 N28 O29
FORMU01_ALERT_2_G  There is a discrepancy between the atom counts in the
            _chemical_formula_sum and the formula from the _atom_site* data.
            Atom count from _chemical_formula_sum:C90 H92 Cl2 Gd6 N28 O29
            Atom count from the _atom_site data:  C90 H86 Cl2 Gd6 N28 O29
CELLZ01_ALERT_1_G Difference between formula and atom_site contents detected.
CELLZ01_ALERT_1_G WARNING: H atoms missing from atom site list. Is this intentional?
           From the CIF: _cell_formula_units_Z    2
           From the CIF: _chemical_formula_sum  C90 H92 Cl2 Gd6 N28 O29
```

TEST: Compare cell contents of formula and atom_site data

atom	Z*formula	cif sites	diff
C	180.00	180.00	0.00
H	184.00	172.00	12.00
Cl	4.00	4.00	0.00
Gd	12.00	12.00	0.00
N	56.00	56.00	0.00
O	58.00	58.00	0.00

```
PLAT002_ALERT_2_G Number of Distance or Angle Restraints on AtSite          8 Note
PLAT003_ALERT_2_G Number of Uiso or Uij Restrained non-H Atoms ...          4 Report
PLAT007_ALERT_5_G Number of Unrefined Donor-H Atoms .............          12 Report
PLAT041_ALERT_1_G Calc. and Reported SumFormula    Strings  Differ     Please Check
PLAT042_ALERT_1_G Calc. and Reported Moiety Formula Strings Differ     Please Check
PLAT083_ALERT_2_G SHELXL Second Parameter in WGHT  Unusually Large      37.03 Why ?
PLAT172_ALERT_4_G The CIF-Embedded .res File Contains DFIX Records          2 Report
PLAT173_ALERT_4_G The CIF-Embedded .res File Contains DANG Records          4 Report
PLAT186_ALERT_4_G The CIF-Embedded .res File Contains ISOR Records          1 Report
PLAT232_ALERT_2_G Hirshfeld Test Diff (M-X)  Gd6      --O27      .        5.3 s.u.
PLAT344_ALERT_2_G Unusual sp?       Angle Range in Solvent/Ion for        C85 Check
PLAT344_ALERT_2_G Unusual sp?       Angle Range in Solvent/Ion for        C83 Check
PLAT606_ALERT_4_G Solvent Accessible VOID(S) in Structure ........          ! Info
PLAT860_ALERT_3_G Number of Least-Squares Restraints ............          30 Note
PLAT933_ALERT_2_G Number of HKL-OMIT Records in Embedded .res File          4 Note
PLAT941_ALERT_3_G Average HKL Measurement Multiplicity ...........        2.8 Low
```

---

0 **ALERT level A** = Most likely a serious problem - resolve or explain
4 **ALERT level B** = A potentially serious problem, consider carefully
18 **ALERT level C** = Check. Ensure it is not caused by an omission or oversight
20 **ALERT level G** = General information/check it is not something unexpected

6 ALERT type 1 CIF construction/syntax error, inconsistent or missing data
26 ALERT type 2 Indicator that the structure model may be wrong or deficient
4 ALERT type 3 Indicator that the structure quality may be low
5 ALERT type 4 Improvement, methodology, query or suggestion
1 ALERT type 5 Informative message, check

It is advisable to attempt to resolve as many as possible of the alerts in all categories. Often the minor alerts point to easily fixed oversights, errors and omissions in your CIF or refinement strategy, so attention to these fine details can be worthwhile. In order to resolve some of the more serious problems it may be necessary to carry out additional measurements or structure refinements. However, the purpose of your study may justify the reported deviations and the more serious of these should normally be commented upon in the discussion or experimental section of a paper or in the "special_details" fields of the CIF. checkCIF was carefully designed to identify outliers and unusual parameters, but every test has its limitations and alerts that are not important in a particular case may appear. Conversely, the absence of alerts does not guarantee there are no aspects of the results needing attention. It is up to the individual to critically assess their own results and, if necessary, seek expert advice.

Publication of your CIF in IUCr journals

A basic structural check has been run on your CIF. These basic checks will be run on all CIFs submitted for publication in IUCr journals (*Acta Crystallographica*, *Journal of Applied Crystallography*, *Journal of Synchrotron Radiation*); however, if you intend to submit to *Acta Crystallographica Section C* or *E* or *IUCrData*, you should make sure that full publication checks are run on the final version of your CIF prior to submission.

Publication of your CIF in other journals

Please refer to the *Notes for Authors* of the relevant journal for any special instructions relating to CIF submission.

PLATON version of 19/02/2022; check.def file version of 19/02/2022

Datablock Gd6 - ellipsoid plot

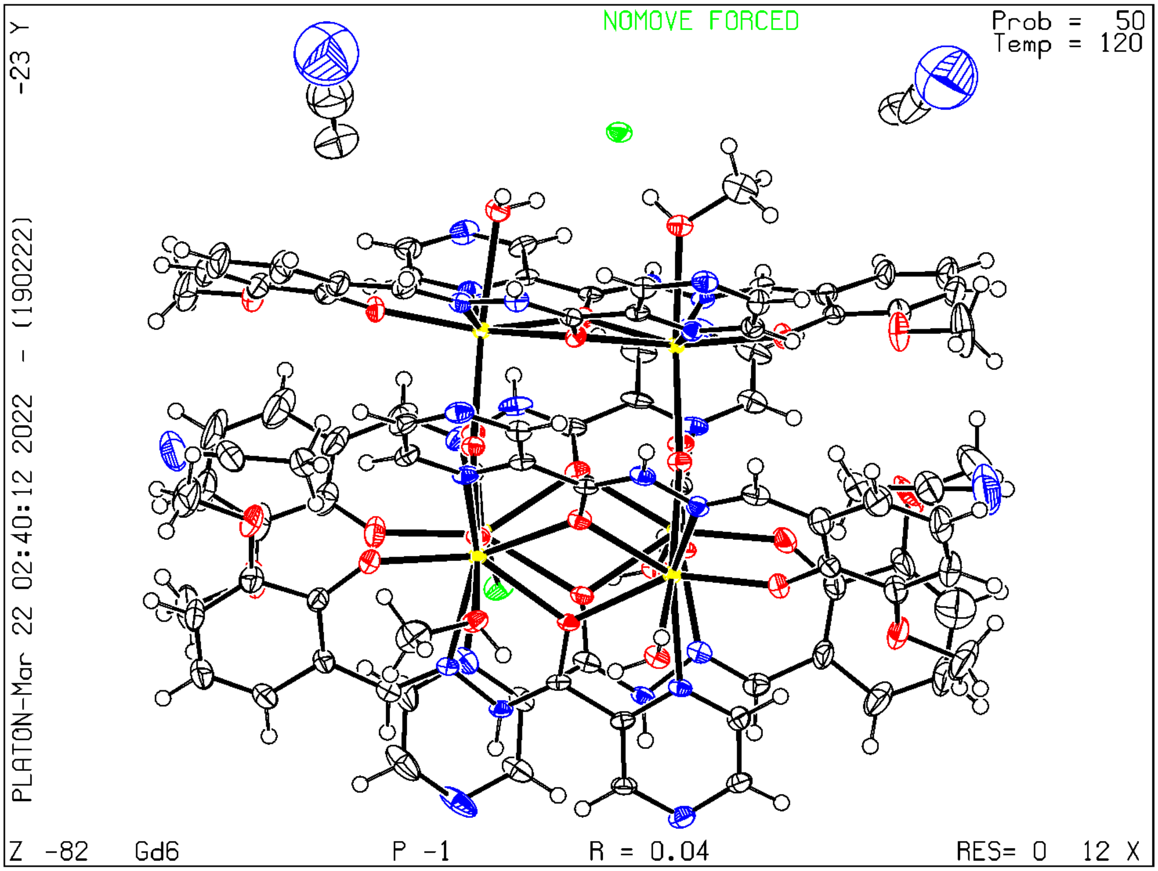

Supplement: Supplementary file 1 [file DataSheet2.PDF]
