# Supplementary material for: Three Gd-based magnetic refrigerant materials with high magnetic entropy: From di-nuclearity to hexa-nuclearity to octa-nuclearity
Source: Front Chem. 2022 Sep 29;10:963203. doi: 10.3389/fchem.2022.963203 (PMC9559567; doi:10.3389/fchem.2022.963203)
Supplement: Supplementary file 2 [file DataSheet4.docx]

Supplementary Material

**Supplementary Table 1.** Selected bond lengths [Å] and angles [°] for **1**.

| Gd1-O2 | 2.233(4) | N3-Gd1-O4 | 95.43(17) | O5-Gd1-O4 | 51.68(15) |
| --- | --- | --- | --- | --- | --- |
| Gd1-O3 | 2.368(4) | N3-Gd1-O5 | 81.76(17) | O5-Gd1-O3 | 82.25(15) |
| Gd1-O4 | 2.460(5) | N3-Gd1-O7 | 96.84(16) | O5-Gd1-O3 | 75.10(15) |
| Gd1-O5 | 2.472(5) | N3-Gd1-O3 | 131.06(15) | O5-Gd1-O2 | 119.83(16) |
| Gd1-O7 | 2.361(4) | O7-Gd1-O5 | 156.82(15) | N3-Gd1-O3 | 64.38(15) |
| Gd1-N3 | 2.460(5) | O7-Gd1-O4 | 150.72(16) | N3-Gd1-O2 | 74.74(17) |
| O7-Gd1-O2 | 81.52(16) | O7-Gd1-O3 | 83.40(15) | O4-Gd1-O3 | 109.68(16) |
| O4-Gd1-O3 | 125.83(15) | O7-Gd1-O3 | 81.39(15) | O3-Gd1-O2 | 134.08(16) |

**Supplementary Table 2.** Selected bond lengths [Å] and angles [°] for **2**.

| Gd1-O21 | 2.420(3) | Gd2-O7 | 2.327(3) | Gd3-O16 | 2.306(3) |
| --- | --- | --- | --- | --- | --- |
| Gd1-O26 | 2.394(3) | Gd2-O10 | 2.324(3) | Gd3-O18 | 2.420(3) |
| Gd1-N1 | 2.569(4) | Gd2-O11 | 2.145(4) | Gd3-O20 | 2.437(3) |
| Gd1-N8 | 2.439(4) | Gd2-O15 | 2.462(3) | Gd3-O21 | 2.418(3) |
| Gd1-O5 | 2.140(4) | Gd2-N16 | 2.476(4) | Gd3-N17 | 2.546(4) |
| Gd1-O4 | 2.302(3) | Gd2-O25 | 2.394(3) | Gd3-N24 | 2.460(4) |
| Gd1-O1 | 2.352(3) | Gd2-N9 | 2.521(4) | Gd3-O13 | 2.335(3) |
| Gd1-O15 | 2.519(3) | Gd2-O20 | 2.442(3) | Gd3-O17 | 2.124(3) |
| Gd4-O24 | 2.548(3) | Gd5-O10 | 2.346(3) | Gd6-O27 | 2.397(3) |
| Gd4-O22 | 2.453(3) | Gd5-O8 | 2.139(4) | Gd6-O24 | 2.465(3) |
| Gd4-O2 | 2.130(4) | Gd5-O7 | 2.303(3) | Gd6-O23 | 2.448(3) |
| Gd4-O1 | 2.315(3) | Gd5-O22 | 2.436(3) | Gd6-O16 | 2.326(3) |
| Gd4-Cl1 | 2.746(1) | Gd5-O23 | 2.416(3) | Gd6-O14 | 2.142(3) |
| Gd4-N5 | 2.544(4) | Gd5-O28 | 2.415(3) | Gd6-N20 | 2.481(4) |
| Gd4-N4 | 2.445(5) | Gd5-N12 | 2.454(4) | Gd6-N21 | 2.515(4) |
| Gd4-O4 | 2.336(3) | Gd5-N13 | 2.550(4) | Gd6-O13 | 2.327(3) |
| O4-Gd1-O15 | 78.77(11) | O7-Gd2-N16 | 127.39(12) | N24-Gd3-N17 | 145.28(13) |
| O4-Gd1-O1 | 67.98(12) | O7-Gd2-N9 | 65.20(12) | O21-Gd3-N24 | 75.92(12) |
| O1-Gd1-N8 | 130.86(13) | O7-Gd2-O25 | 78.42(12) | O21-Gd3-N17 | 131.61(12) |
| O1-Gd1-N1 | 64.95(13) | O7-Gd2-O20 | 108.66(11) | O21-Gd3-O20 | 54.58(11) |
| O1-Gd1-O26 | 79.01(12) | O7-Gd2-O15 | 85.22(11) | O21-Gd3-O18 | 155.75(11) |
| O4-Gd1-O26 | 80.03(12) | N16-Gd2-N9 | 147.38(13) | O18-Gd3-N24 | 79.85(12) |
| O4-Gd1-N1 | 129.79(13) | O10-Gd2-O7 | 66.62(11) | O18-Gd3-N17 | 71.27(12) |
| O4-Gd1-N8 | 65.97(13) | O10-Gd2-O15 | 111.74(11) | O18-Gd3-O20 | 149.01(11) |
| O5-Gd1-O1 | 148.05(14) | O10-Gd2-O20 | 77.59(11) | O17-Gd3-N24 | 75.77(14) |
| O5-Gd1-O4 | 141.25(13) | O10-Gd2-O25 | 85.98(12) | O17-Gd3-N17 | 81.96(13) |
| O5-Gd1-O15 | 92.89(14) | O10-Gd2-N9 | 130.66(12) | O17-Gd3-O21 | 91.65(12) |
| O5-Gd1-O21 | 96.42(14) | O10-Gd2-N16 | 65.01(13) | O20-Gd3-N17 | 77.74(11) |
| O5-Gd1-O26 | 92.43(15) | O11-Gd2-O7 | 149.25(12) | O20-Gd3-N24 | 128.99(12) |
| O5-Gd1-N1 | 83.11(15) | O1-Gd2-O10 | 139.62(13) | O17-Gd3-O20 | 92.69(12) |
| O5-Gd1-N8 | 75.29(14) | O11-Gd2-O15 | 94.17(13) | O17-Gd3-O18 | 83.24(13) |
| O15-Gd1-N1 | 132.84(11) | O11-Gd2-O20 | 95.34(13) | O17-Gd3-O16 | 140.68(12) |
| O21-Gd1-O15 | 53.62(10) | O11-Gd2-O25 | 86.89(13) | O17-Gd3-O13 | 146.47(12) |
| O21-Gd1-N1 | 80.04(11) | O11-Gd2-N9 | 85.00(13) | O16-Gd3-N24 | 65.20(13) |
| O21-Gd1-N8 | 130.57(12) | O11-Gd2-N16 | 74.62(14) | O16-Gd3-N17 | 128.98(12) |
| O26-Gd1-O15 | 152.15(11) | O15-Gd2-N9 | 74.17(11) | O16-Gd3-O21 | 83.69(11) |
| O26-Gd1-O21 | 152.24(11) | O15-Gd2-N16 | 131.54(12) | O16-Gd3-O20 | 115.09(11) |
| O26-Gd1-N1 | 74.96(12) | O20-Gd2-N9 | 128.04(11) | O16-Gd3-O18 | 85.38(12) |
| O26-Gd1-N8 | 77.15(12) | O20-Gd2-N16 | 79.84(12) | O16-Gd3-O13 | 67.07(11) |
| N8-Gd1-O15 | 77.83(11) | O25-Gd2-O1 | 148.73(11) | O13-Gd3-N24 | 129.60(13) |
| N8-Gd1-N1 | 143.69(13) | O25-Gd2-O20 | 157.14(11) | O13-Gd3-N17 | 64.97(12) |
| O1-Gd1-O21 | 79.62(11) | O25-Gd2-N9 | 74.81(12) | O13-Gd3-O21 | 113.93(11) |
| O1-Gd1-O15 | 109.07(11) | O25-Gd2-N16 | 78.85(12) | O13-Gd3-O20 | 85.82(11) |
| O4-Gd1-O21 | 107.87(11) | O20-Gd2-O15 | 53.94(10) | O13-Gd3-O18 | 81.17(12) |
| O4-Gd4-O24 | 112.03(11) | N12-Gd5-N13 | 147.27(14) | O14-Gd6-N21 | 84.99(14) |
| N5-Gd4-O24 | 133.04(12) | O28-Gd5-N13 | 71.35(12) | N20-Gd6-N21 | 145.77(13) |
| N5-Gd4-Cl1 | 76.34(10) | O28-Gd5-N12 | 81.69(13) | O27-Gd6-N21 | 73.38(12) |
| N4-Gd4-N5 | 144.55(14) | O28-Gd5-O23 | 148.51(11) | O27-Gd6-N20 | 78.17(12) |
| N4-Gd4-O24 | 75.71(12) | O28-Gd5-O22 | 156.76(11) | O27-Gd6-O24 | 146.21(11) |
| N4-Gd4-O22 | 127.78(12) | O7-Gd5-O10 | 66.64(11) | O27-Gd6-O23 | 159.31(12) |
| N4-Gd4-Cl1 | 76.02(10) | O7-Gd5-O22 | 80.54(11) | O24-Gd6-N21 | 73.65(12) |
| O24-Gd4-Cl1 | 150.47(8) | O7-Gd5-O23 | 115.06(11) | O24-Gd6-N20 | 135.35(12) |
| O22-Gd4-N5 | 81.28(12) | O7-Gd5-O28 | 85.64(12) | O23-Gd6-N21 | 127.29(12) |
| O22-Gd4-O24 | 52.86(10) | O7-Gd5-N12 | 65.20(13) | O23-Gd6-N20 | 82.60(12) |
| O22-Gd4-Cl1 | 156.17(8) | O7-Gd5-N13 | 128.56(13) | O23-Gd6-O24 | 53.96(11) |
| O4-Gd4-N5 | 65.30(13) | O8-Gd5-O7 | 140.49(12) | O16-Gd6-N21 | 64.88(13) |
| O4-Gd4-N4 | 130.22(14) | O8-Gd5-O10 | 146.77(12) | O16-Gd6-N20 | 126.50(13) |
| O4-Gd4-O22 | 82.79(11) | O8-Gd5-O22 | 95.86(13) | O16-Gd6-O27 | 77.39(12) |
| O4-Gd4-Cl1 | 80.56(8) | O8-Gd5-O23 | 93.02(13) | O16-Gd6-O24 | 82.19(11) |
| O2-Gd4-N5 | 84.25(15) | O8-Gd5-O28 | 83.03(13) | O16-Gd6-O23 | 108.79(11) |
| O2-Gd4-N4 | 75.34(16) | O8-Gd5-N12 | 75.74(14) | O16-Gd6-O13 | 66.87(11) |
| O2-Gd4-O24 | 88.29(13) | O8-Gd5-N13 | 82.73(14) | O13-Gd6-O23 | 78.44(11) |
| O2-Gd4-O22 | 93.06(14) | O10-Gd5-O22 | 110.21(11) | O14-Gd6-N20 | 74.62(14) |
| O2-Gd4-O4 | 149.56(14) | O10-Gd5-O23 | 86.09(11) | O14-Gd6-O27 | 86.67(13) |
| O2-Gd4-O1 | 141.13(14) | O10-Gd5-O28 | 80.74(12) | O14-Gd6-O24 | 97.63(13) |
| O2-Gd4-Cl1 | 92.72(11) | O10-Gd5-N12 | 129.60(13) | O14-Gd6-O23 | 95.59(12) |
| O1-Gd4-N5 | 131.50(13) | O1-Gd5-N13 | 64.66(12) | O14-Gd6-O16 | 148.79(13) |
| O1-Gd4-N4 | 66.23(14) | O22-Gd5-N12 | 75.59(12) | O14-Gd6-O13 | 139.38(12) |
| O1-Gd4-O24 | 77.18(11) | O22-Gd5-N13 | 131.69(12) | O13-Gd6-N21 | 130.56(13) |
| O1-Gd4-O22 | 105.58(11) | O23-Gd5-O22 | 54.62(11) | O13-Gd6-N20 | 64.78(12) |
| O1-Gd4-O4 | 68.06(11) | O23-Gd5-N12 | 127.71(13) | O13-Gd6-O27 | 86.45(12) |
| O1-Gd4-Cl1 | 83.79(8) | O23-Gd5-N13 | 77.16(12) | O13-Gd6-O24 | 109.86(11) |

**Supplementary Table 3.** Selected bond lengths [Å] and angles [°] for **3**.

| Gd1-N19 | 2.453(5) | Gd3-O4 | 2.360(3) | Gd5-O20 | 2.208(3) | Gd7-O24 | 2.348(3) |
| --- | --- | --- | --- | --- | --- | --- | --- |
| Gd1-N1 | 2.645(4) | Gd3-O5 | 2.201(4) | Gd5-O19 | 2.347(3) | Gd7-O33 | 2.327(3) |
| Gd1-O44 | 2.351(3) | Gd3-O16 | 2.352(3) | Gd5-O9 | 2.375(3) | Gd7-O37 | 2.360(3) |
| Gd1-O31 | 2.327(3) | Gd3-O27 | 2.311(3) | Gd5-O30 | 2.336(3) | Gd7-O12 | 2.354(3) |
| Gd1-O25 | 2.326(3) | Gd3-O34 | 2.311(3) | Gd5-O36 | 2.298(3) | Gd7-O11 | 2.227(3) |
| Gd1-O14 | 2.214(4) | Gd3-O39 | 2.364(3) | Gd5-O41 | 2.356(3) | Gd7-N16 | 2.458(4) |
| Gd1-O13 | 2.353(4) | Gd3-N7 | 2.464(4) | Gd5-N11 | 2.634(4) | Gd7-N31 | 2.613(4) |
| Gd1-O1 | 2.360(4) | Gd3-N21 | 2.627(4) | Gd5-N27 | 2.467(4) | Gd7-O28 | 2.301(3) |
| Gd2-N4 | 2.511(5) | Gd4-N25 | 2.618(4) | Gd6-O9 | 2.426(3) | Gd8-O29 | 2.413(3) |
| Gd2-N41 | 2.675(5) | Gd4-O16 | 2.398(3) | Gd6-O12 | 2.461(3) | Gd8-O24 | 2.391(3) |
| Gd2-O38 | 2.450(4) | Gd4-O17 | 2.247(3) | Gd6-O28 | 2.388(3) | Gd8-O31 | 2.394(3) |
| Gd2-O32 | 2.382(3) | Gd4-O19 | 2.468(3) | Gd6-O30 | 2.517(3) | Gd8-O33 | 2.491(3) |
| Gd2-O27 | 2.375(3) | Gd4-O26 | 2.388(3) | Gd6-O35 | 2.390(3) | Gd8-O43 | 2.421(3) |
| Gd2-O25 | 2.492(3) | Gd4-O36 | 2.383(3) | Gd6-O42 | 2.399(3) | Gd8-N17 | 2.664(4) |
| Gd2-O4 | 2.488(4) | Gd4-O40 | 2.407(3) | Gd6-N9 | 2.507(4) | Gd8-N29 | 2.493(4) |
| Gd2-O2 | 2.233(3) | Gd4-N24 | 2.509(4) | Gd6-N13 | 2.689(4) | Gd8-O23 | 2.230(3) |
| Gd2-O1 | 2.406(4) | Gd4-O34 | 2.520(3) | Gd6-O8 | 2.234(3) | Gd8-O13 | 2.481(3) |
| O25-Gd1-O1 | 69.15(12) | O4-Gd2-O25 | 100.86(11) | O27-Gd3-O4 | 66.86(12) | O34-Gd4-N25 | 137.43(12) |
| O25-Gd1-O13 | 137.97(11) | O4-Gd2-N4 | 127.57(15) | O27-Gd3-O16 | 78.16(11) | O36-Gd4-O16 | 114.34(10) |
| O25-Gd1-O31 | 73.25(11) | O4-Gd2-N41 | 60.99(15) | O27-Gd3-O34 | 74.71(11) | O36-Gd4-O19 | 64.69(10) |
| O25-Gd1-O44 | 75.68(11) | O25-Gd2-N4 | 129.28(15) | O27-Gd3-O39 | 82.59(13) | O36-Gd4-O26 | 78.64(11) |
| O25-Gd1-N1 | 130.00(14) | O25-Gd2-N41 | 135.86(12) | O27-Gd3-N7 | 130.99(16) | O36-Gd4-O34 | 52.29(10) |
| O25-Gd1-N19 | 140.94(14) | O27-Gd2-O1 | 114.68(12) | O27-Gd3-N21 | 102.91(12) | O36-Gd4-O40 | 101.58(13) |
| O31-Gd1-O1 | 78.09(12) | O27-Gd2-O4 | 63.86(11) | N7-Gd3-N21 | 78.39(14) | O36-Gd4-N24 | 152.76(12) |
| O31-Gd1-O13 | 67.25(11) | O27-Gd2-O25 | 52.82(11) | O39-Gd3-N21 | 151.82(11) | O36-Gd4-N25 | 126.87(12) |
| O31-Gd1-O44 | 81.18(11) | O27-Gd2-O32 | 78.93(11) | O39-Gd3-N7 | 77.42(14) | N24-Gd4-N25 | 73.21(13) |
| O31-Gd1-N1 | 109.23(13) | O27-Gd2-O38 | 102.92(17) | O34-Gd3-N21 | 132.91(11) | N24-Gd4-O34 | 128.42(11) |
| O44-Gd1-O1 | 142.99(12) | O27-Gd2-N4 | 157.67(13) | O34-Gd3-N7 | 138.88(14) | O40-Gd4-N25 | 68.72(14) |
| O44-Gd1-O13 | 84.52(12) | O27-Gd2-N41 | 124.68(15) | O34-Gd3-O39 | 75.27(11) | O40-Gd4-N24 | 103.39(15) |
| O44-Gd1-N1 | 153.74(15) | O32-Gd2-O1 | 71.21(12) | O34-Gd3-O16 | 69.7(1) | O40-Gd4-O34 | 70.39(12) |
| O44-Gd1-N19 | 77.92(14) | O32-Gd2-O4 | 128.70(11) | O34-Gd3-O4 | 137.09(12) | O40-Gd4-O19 | 81.06(12) |
| N19-Gd1-N1 | 77.09(16) | O32-Gd2-O25 | 80.84(11) | O16-Gd3-N21 | 63.98(11) | O16-Gd4-O19 | 153.26(11) |
| O14-Gd1-N19 | 74.51(18) | O32-Gd2-O38 | 137.99(16) | O16-Gd3-N7 | 137.84(14) | O16-Gd4-O34 | 65.57(10) |
| O14-Gd1-N1 | 80.04(15) | O32-Gd2-N4 | 79.73(14) | O16-Gd3-O39 | 143.33(11) | O16-Gd4-O40 | 72.90(12) |
| O14-Gd1-O44 | 100.90(14) | O32-Gd2-N41 | 142.79(12) | O16-Gd3-O4 | 117.92(12) | O16-Gd4-N24 | 63.88(12) |
| O14-Gd1-O31 | 154.73(16) | O38-Gd2-O4 | 86.30(19) | O5-Gd3-N21 | 85.04(13) | O16-Gd4-N25 | 111.88(12) |
| O14-Gd1-O25 | 82.80(15) | O38-Gd2-O25 | 68.78(12) | O5-Gd3-N7 | 73.74(17) | O17-Gd4-O16 | 130.81(11) |
| O14-Gd1-O13 | 137.92(15) | O38-Gd2-N4 | 97.30(19) | O5-Gd3-O39 | 101.73(14) | O17-Gd4-O19 | 74.44(10) |
| O14-Gd1-O1 | 86.05(15) | O38-Gd2-N41 | 70.10(15) | O5-Gd3-O34 | 82.40(13) | O17-Gd4-O26 | 75.94(11) |
| O13-Gd1-N19 | 65.84(15) | N4-Gd2-N41 | 71.26(18) | O5-Gd3-O16 | 84.31(13) | O17-Gd4-O34 | 142.91(11) |
| O13-Gd1-N1 | 78.02(14) | O2-Gd2-O25 | 145.23(11) | O5-Gd3-O4 | 138.12(13) | O17-Gd4-O36 | 94.60(11) |
| O13-Gd1-O1 | 114.40(13) | O2-Gd2-O4 | 74.74(13) | O4-Gd3-N21 | 75.73(12) | O17-Gd4-O40 | 141.23(12) |
| O1-Gd1-N19 | 138.21(14) | O2-Gd2-O1 | 128.07(13) | O4-Gd3-N7 | 66.19(16) | O17-Gd4-N24 | 72.29(12) |
| O1-Gd1-N1 | 63.12(15) | O2-Gd2-O38 | 142.99(14) | O4-Gd3-O39 | 81.42(12) | O17-Gd4-N25 | 73.35(13) |
| O31-Gd1-N19 | 129.91(15) | O1-Gd2-N41 | 113.52(16) | O5-Gd3-O27 | 154.86(12) | O19-Gd4-O34 | 100.27(10) |
| O9-Gd5-N11 | 63.65(11) | O8-Gd6-O9 | 127.97(11) | O12-Gd7-O37 | 81.98(11) | O23-Gd8-O13 | 75.16(12) |
| O9-Gd5-N27 | 135.47(12) | O8-Gd6-O12 | 73.88(11) | O11-Gd7-N31 | 84.84(13) | O23-Gd8-O24 | 130.32(11) |
| O19-Gd5-O9 | 118.71(11) | O8-Gd6-O28 | 95.66(11) | O11-Gd7-N16 | 74.57(13) | O23-Gd8-O29 | 78.21(11) |
| O19-Gd5-O41 | 80.74(11) | O8-Gd6-O30 | 143.74(10) | O11-Gd7-O37 | 103.62(13) | O23-Gd8-O31 | 94.94(12) |
| O19-Gd5-N11 | 74.98(12) | O8-Gd6-O35 | 76.12(11) | O11-Gd7-O33 | 83.74(11) | O23-Gd8-O33 | 144.72(11) |
| O19-Gd5-N27 | 66.16(14) | O8-Gd6-O42 | 142.16(11) | O11-Gd7-O28 | 154.16(11) | O23-Gd8-O43 | 140.96(11) |
| O20-Gd5-O9 | 84.22(11) | O8-Gd6-N9 | 71.95(12) | O11-Gd7-O24 | 82.04(12) | O23-Gd8-N17 | 74.79(13) |
| O20-Gd5-O19 | 139.47(11) | O8-Gd6-N13 | 73.38(11) | O11-Gd7-O12 | 137.42(11) | O23-Gd8-N29 | 72.00(13) |
| O20-Gd5-O30 | 80.37(11) | O9-Gd6-O12 | 156.97(10) | N16-Gd7-N31 | 80.06(13) | O24-Gd8-O13 | 152.85(11) |
| O20-Gd5-O36 | 152.57(11) | O9-Gd6-O30 | 66.15(10) | O37-Gd7-N31 | 151.96(12) | O24-Gd8-O29 | 70.82(11) |
| O20-Gd5-O41 | 101.01(12) | O9-Gd6-N9 | 63.32(11) | O37-Gd7-N16 | 76.68(12) | O24-Gd8-O31 | 115.04(10) |
| O20-Gd5-N11 | 88.86(12) | O9-Gd6-N13 | 113.51(11) | O33-Gd7-N31 | 133.95(12) | O24-Gd8-O33 | 66.92(10) |
| O20-Gd5-N27 | 74.17(14) | O12-Gd6-O30 | 101.17(10) | O33-Gd7-N16 | 137.92(12) | O24-Gd8-O43 | 69.22(11) |
| O30-Gd5-O9 | 69.89(10) | O12-Gd6-N9 | 128.08(11) | O33-Gd7-O37 | 74.00(11) | O24-Gd8-N17 | 111.10(13) |
| O30-Gd5-O19 | 137.07(10) | O12-Gd6-N13 | 61.63(11) | O33-Gd7-O24 | 70.35(11) | O24-Gd8-N29 | 63.93(12) |
| O30-Gd5-O41 | 74.73(10) | O28-Gd6-O9 | 114.18(10) | O33-Gd7-O12 | 136.83(11) | O29-Gd8-O13 | 131.34(11) |
| O30-Gd5-N11 | 133.11(11) | O28-Gd6-O12 | 64.56(10) | O28-Gd7-N31 | 100.33(12) | O29-Gd8-O33 | 81.48(10) |
| O30-Gd5-N27 | 139.98(13) | O28-Gd6-O30 | 52.36(10) | O28-Gd7-N16 | 131.19(12) | O29-Gd8-O43 | 137.50(11) |
| O36-Gd5-O9 | 78.11(10) | O28-Gd6-O35 | 78.05(11) | O28-Gd7-O37 | 83.73(11) | O29-Gd8-N17 | 144.59(12) |
| O36-Gd5-O19 | 67.95(10) | O28-Gd6-O42 | 103.00(12) | O28-Gd7-O33 | 74.43(11) | O29-Gd8-N29 | 77.02(12) |
| O36-Gd5-O30 | 73.94(11) | O28-Gd6-N9 | 155.41(12) | O28-Gd7-O24 | 77.75(11) | O31-Gd8-O13 | 64.20(11) |
| O36-Gd5-O41 | 81.56(11) | O28-Gd6-N13 | 126.06(11) | O28-Gd7-O12 | 67.62(10) | O31-Gd8-O29 | 78.58(11) |
| O36-Gd5-N11 | 101.62(12) | O35-Gd6-N13 | 142.45(11) | O24-Gd7-N31 | 63.93(11) | O3- Gd8-O33 | 52.61(10) |
| O36-Gd5-N27 | 132.68(14) | O35-Gd6-N9 | 78.36(12) | O24-Gd7-N16 | 138.53(12) | O3- Gd8-O43 | 105.94(11) |
| O41-Gd5-O9 | 142.82(10) | O35-Gd6-O42 | 139.65(11) | O24-Gd7-O37 | 143.05(11) | O31-Gd8-N17 | 125.91(13) |
| O41-Gd5-N11 | 152.00(12) | O35-Gd6-O30 | 80.19(10) | O24-Gd7-O12 | 118.62(10) | O31-Gd8-N29 | 154.21(12) |
| O41-Gd5-N27 | 80.38(12) | O35-Gd6-O12 | 128.5(1) | O12-Gd7-N31 | 74.24(11) | O33-Gd8-N17 | 132.96(12) |
| N27-Gd5-N11 | 77.22(13) | O35-Gd6-O9 | 70.26(10) | O12-Gd7-N16 | 65.66(12) | O33-Gd8-N29 | 130.42(11) |

**Supplementary Table 4.** Continuous shape measures calculations (CShM) for Gd ion in **1**.

| shape | HP-7  (*D*_7h_) | HPY-7  (*C*_6v_) | PBPY-7  (*D*_5h_) | COC-7  (*C*_3v_) | CTPR-7  (*C*_2v_) | JPBPY-7  (*D*_5h_) | JETPY-7  (*C*_3v_) |
| --- | --- | --- | --- | --- | --- | --- | --- |
| Gd | 28.821 | 17.587 | 8.533 | 4.889 | 4.655 | 11.078 | 15.070 |

*HP-7 = Heptagon; HPY-7 = Hexagonal pyramid; PBPY-7 = Pentagonal bipyramid; COC-7 = Capped octahedron; CTPR-7 =Capped trigonal prism; JPBPY-7 = Johnson pentagonal bipyramid J13; JETPY-7 = Johnson elongated triangular pyramid J7.

**Supplementary Table 5.** Continuous shape measures calculations (CShM) for Gd ions in **2**-**3**.

| shape | OP-8  (*D*_8h_) | HPY-8  (*C*_7v_) | HBPY-8  (*D*_6h_) | CU-8  (*O*_h_) | SAPR-8  (*D*_4d_) | TDD-8  (*D*_2d_) | JGBF-8  (*D*_2d_) | JETBPY-8  (*D*_3h_) | JBTP-8  (*C*_2v_) | BTPR-8  (*C*_2v_) | JSD-8  (*D*_2d_) | TT-8  (*T*_d_) | ETBPY-8  (*D*_3h_) |
| --- | --- | --- | --- | --- | --- | --- | --- | --- | --- | --- | --- | --- | --- |
| Gd1 in **2** | 32.263 | 22.505 | 16.606 | 13.431 | 3.908 | 3.037 | 14.094 | 26.326 | 2.801 | 2.723 | 4.350 | 13.725 | 24.205 |
| Gd2 in **2** | 32.702 | 22.181 | 15.096 | 13.226 | 4.948 | 3.498 | 11.964 | 24.178 | 3.088 | 3.027 | 4.736 | 13.708 | 21.786 |
| Gd3 in **2** | 32.189 | 22.112 | 16.571 | 14.375 | 5.011 | 3.631 | 13.153 | 23.733 | 2.819 | 2.672 | 4.788 | 14.744 | 21.298 |
| Gd4 in **2** | 32.164 | 22.975 | 16.655 | 13.762 | 4.139 | 2.964 | 14.420 | 27.494 | 3.526 | 3.199 | 4.788 | 14.381 | 25.120 |
| Gd5 in **2** | 32.765 | 21.988 | 16.455 | 14.748 | 5.303 | 3.500 | 12.864 | 23.479 | 3.065 | 2.936 | 4.737 | 15.128 | 21.157 |
| Gd6 in **2** | 33.306 | 22.584 | 14.538 | 12.254 | 4.937 | 3.156 | 12.157 | 24.224 | 3.130 | 3.095 | 4.983 | 12.762 | 21.607 |
| Gd1 in **3** | 29.382 | 23.929 | 14.248 | 9.807 | 2.232 | 2.038 | 12.908 | 27.462 | 2.246 | 1.757 | 4.191 | 10.303 | 23.759 |
| Gd3 in **3** | 30.071 | 24.415 | 14.254 | 10.388 | 2.128 | 1.900 | 12.387 | 28.457 | 2.352 | 1.952 | 3.708 | 10.896 | 24.222 |
| Gd5 in **3** | 30.198 | 24.405 | 14.856 | 11.129 | 2.315 | 1.698 | 12.693 | 28.202 | 2.391 | 2.050 | 3.494 | 11.647 | 24.538 |
| Gd7 in **3** | 30.207 | 23.717 | 13.697 | 10.397 | 2.082 | 1.754 | 11.824 | 28.564 | 2.531 | 2.207 | 3.579 | 10.945 | 24.161 |

* OP-8 = Octagon; HPY-8 = Heptagonal pyramid; HBPY-8 = Hexagonal bipyramid; CU-8 = Cube; SAPR-8 = Square antiprism; TDD-8 = Triangular dodecahedron; JGBF-8 = Johnson-Gyrobifastigium; JETBPY-8 = Johnson-Elongated triangular bipyramid (J14); JBTP-8 = Johnson-Biaugmented trigonal prism (J50); BTPR-8 = Biaugmented trigonal prism; JSD-8 = Snub disphenoid (J84); TT-8 = Triakis tetrahedron; ETBPY-8 = Elongated trigonal bipyramid.

**Supplementary Table 6.** Continuous shape measures calculations (CShM) for Gd ions in **3**.

| shape | EP-9  (*D*_8h_) | OPY-9  (*C*_7v_) | HBPY-9  (*D*_6h_) | JTC-9  (*O*_h_) | JCCU-9  (*D*_4d_) | CCU-9  (*D*_2d_) | JCSAPR-9  (*D*_2d_) | CSAPR-9(*D*_3h_) | JTCTPR-9(*C*_2v_) | TCTPR-9(*C*_2v_) | JTDIC-9  (*D*_2d_) | HH-9  (*T*_d_) | MFF-9  (*D*_3h_) |
| --- | --- | --- | --- | --- | --- | --- | --- | --- | --- | --- | --- | --- | --- |
| Gd2 in **3** | 32.414 | 22.160 | 16.631 | 13.439 | 7.305 | 6.273 | 4.837 | 3.581 | 5.360 | 4.590 | 10.483 | 5.860 | 2.311 |
| Gd4 in **3** | 31.397 | 22.204 | 16.302 | 13.811 | 7.995 | 6.917 | 4.312 | 3.052 | 5.409 | 3.922 | 10.670 | 6.498 | 1.754 |
| Gd6 in **3** | 31.976 | 21.941 | 16.604 | 13.494 | 7.222 | 6.325 | 4.670 | 3.472 | 5.267 | 4.536 | 10.487 | 6.032 | 2.147 |
| Gd8 in **3** | 33.056 | 22.353 | 16.858 | 13.196 | 7.977 | 6.988 | 4.196 | 2.922 | 4.875 | 4.006 | 10.085 | 7.103 | 1.695 |

* EP-9 = Enneagon; OPY-9 = Octagonal pyramid; HBPY-9 = Heptagonal bipyramid; JTC-9 = Triangular cupola (J3) = trivacant cuboctahedron; JCCU-9 = Capped cube (Elongated square pyramid, J8); CCU-9 = Capped cube; JCSAPR-9 = Capped sq. antiprism (Gyroelongated square pyramid J10); CSAPR-9 = Capped square antiprism); JTCTPR-9 = Tricapped trigonal prism (J51); TCTPR-9 = Tricapped trigonal prism; JTDIC-9 = Tridiminished icosahedron (J63); HH-9 = Hula-hoop; MFF-9 = Muffin.

**Supplementary Table 7.** The resulted parameters from the fitting of Curie-Weiss law.

|  | **1** | **2** | **3** |
| --- | --- | --- | --- |
| *C* (cm^3^ mol^-1^ K) | 15.72 | 47.10 | 62.89 |
| *θ* (K) | -1.44 | -1.33 | -0.45 |


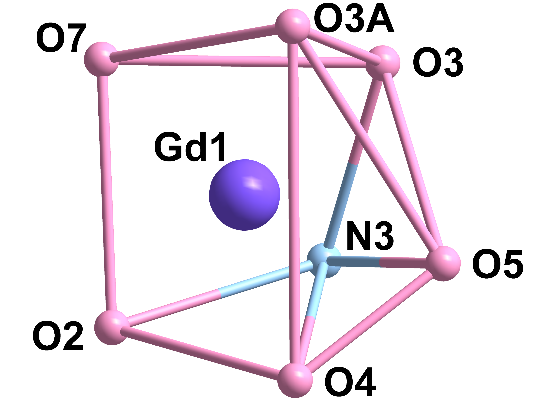


**Supplementary Figure 1.** Coordination geometry of Ln ion in complex **1**. Color codes: Gd, purple; O, pink; N, blue. Symmetric code: A, 1-x, 1-y, 1-z.

**
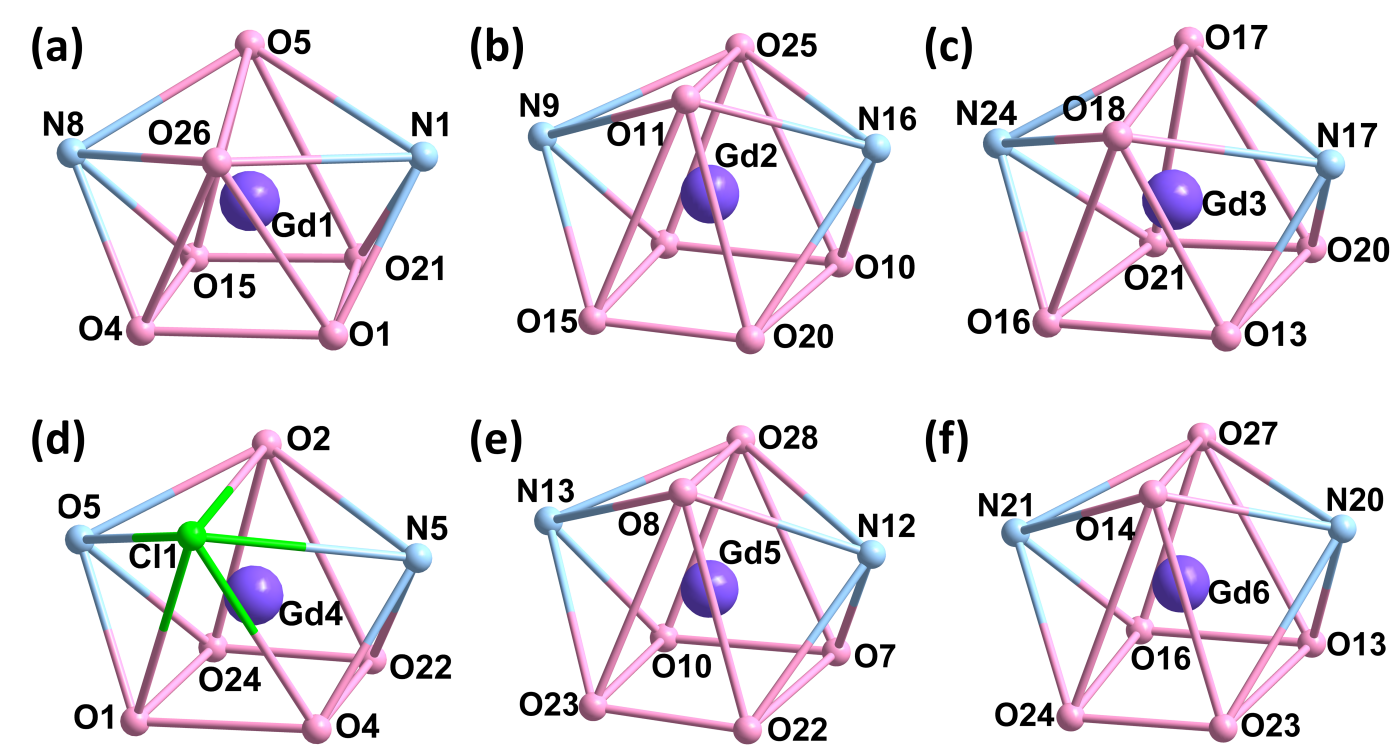
**

**Supplementary Figure 2.** Coordination geometry of Ln ion in complex **2**. Color codes: Gd, purple; O, pink; N, blue.


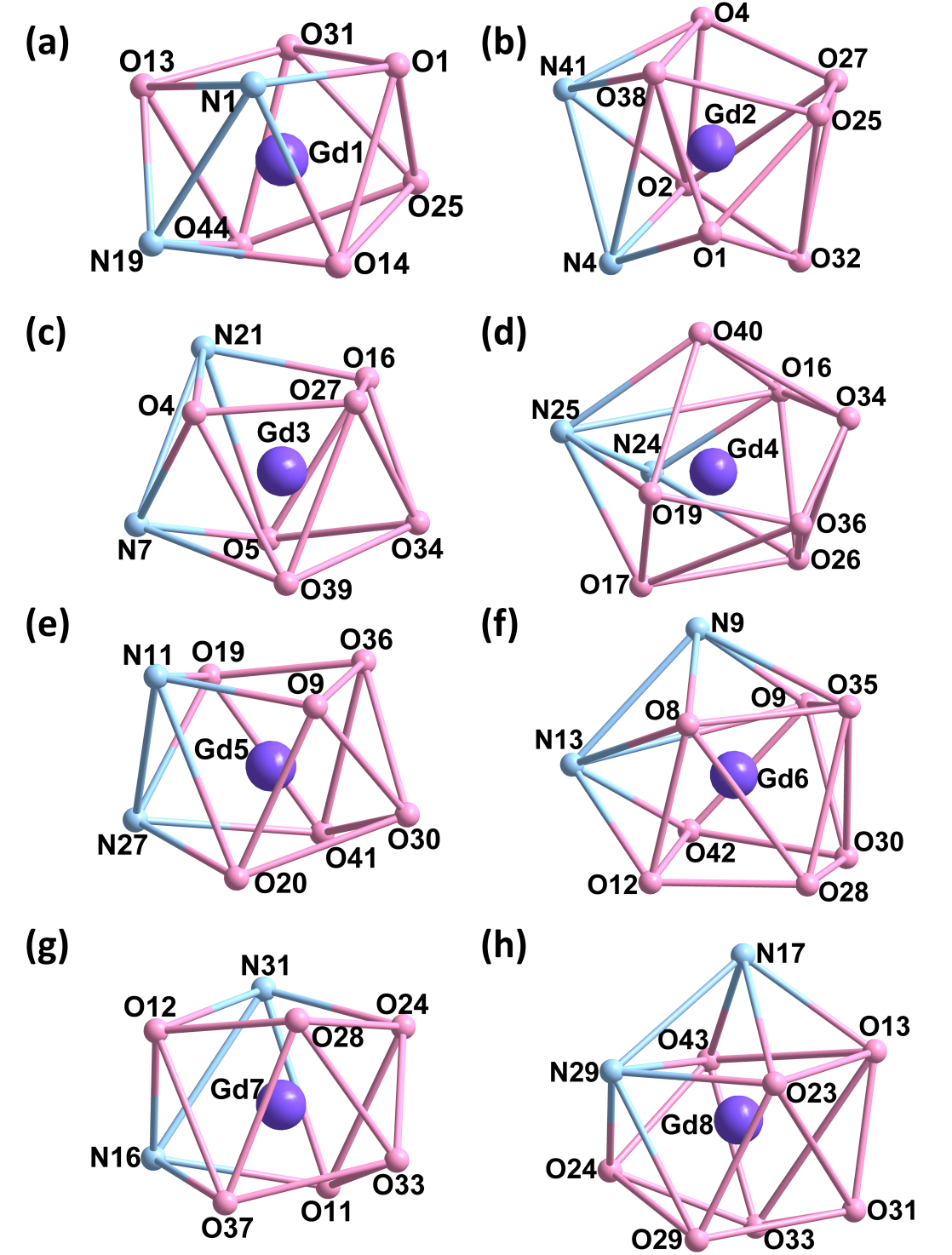


**Supplementary Figure 3.** Coordination geometry of Ln ion in complex **3**. Color codes: Gd, purple; O, pink; N, blue.


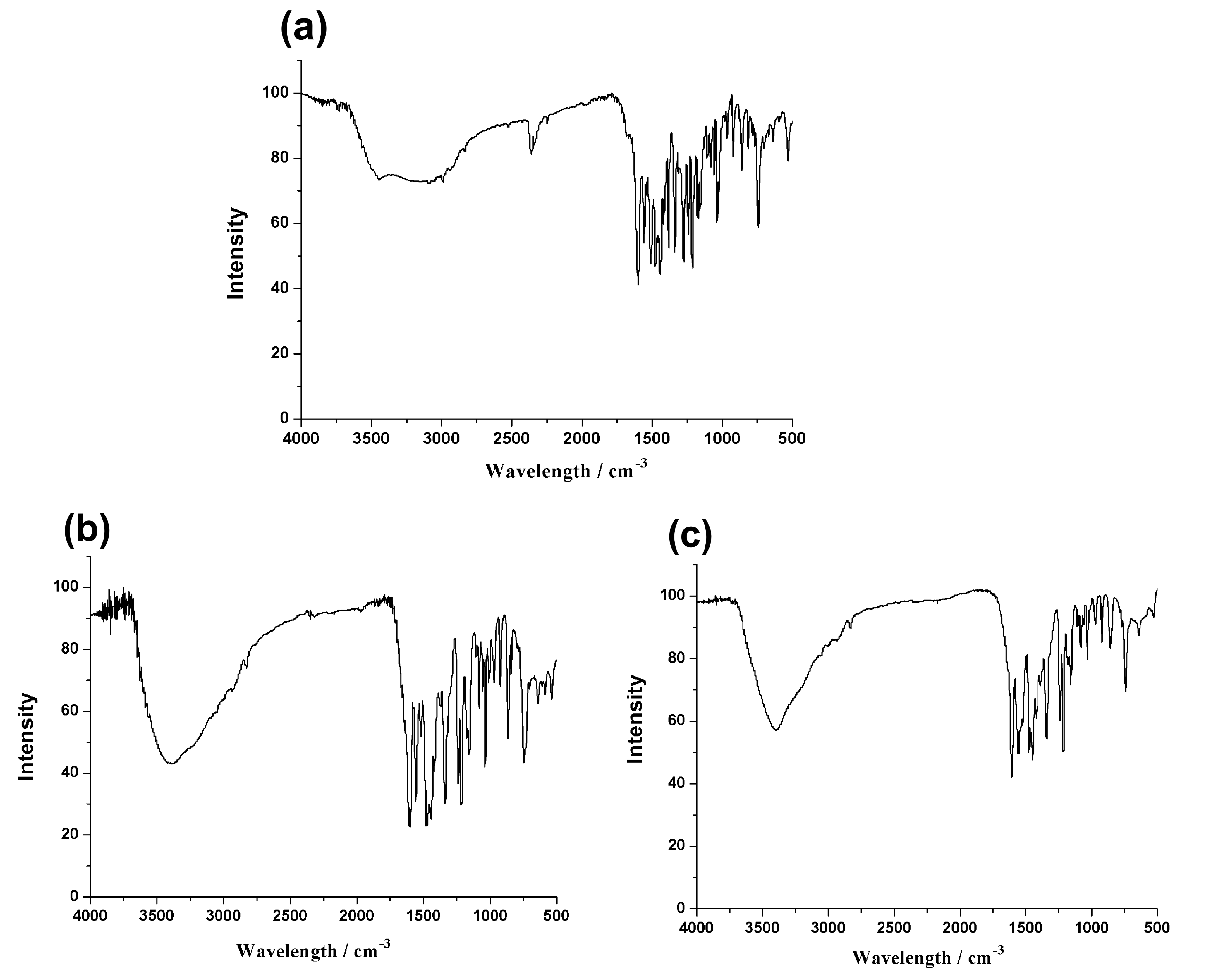


**Supplementary Figure 4.** FT-IR spectra of complexes **1-3** (a-c).


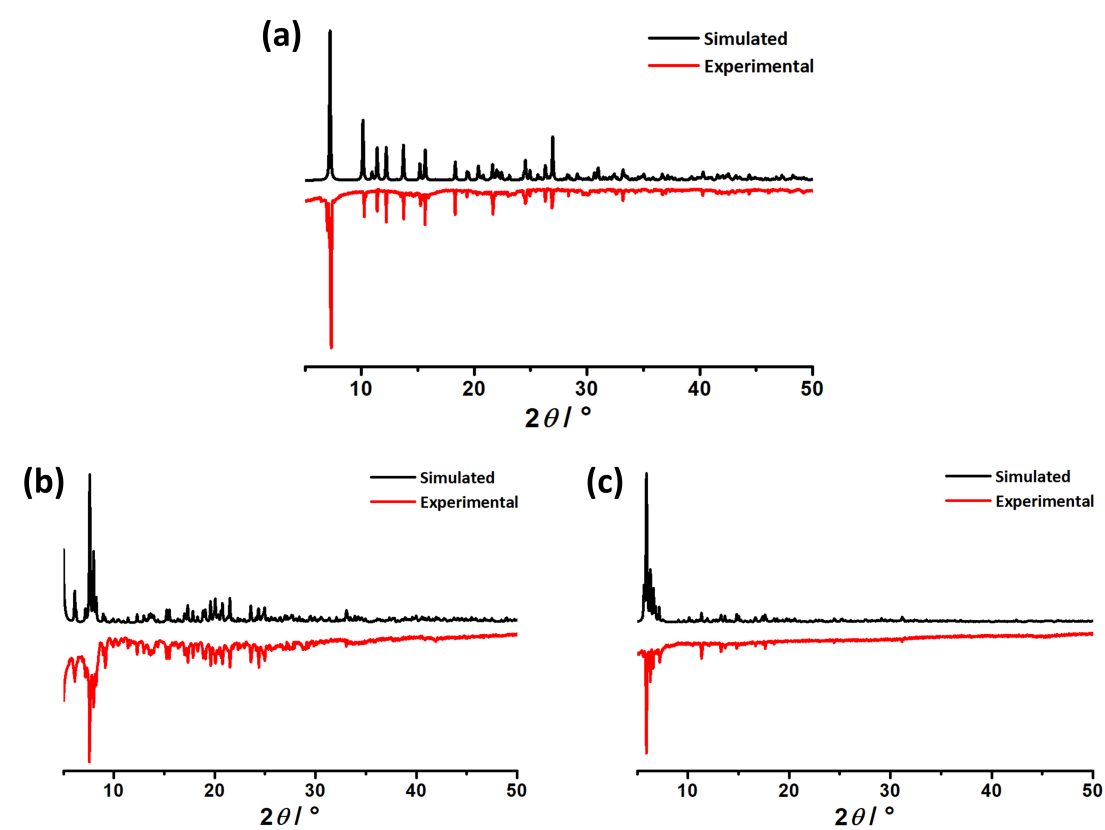


**Supplementary Figure 5.** Experimental and simulated powder X-ray diffraction for **1**-**3** (a-c).


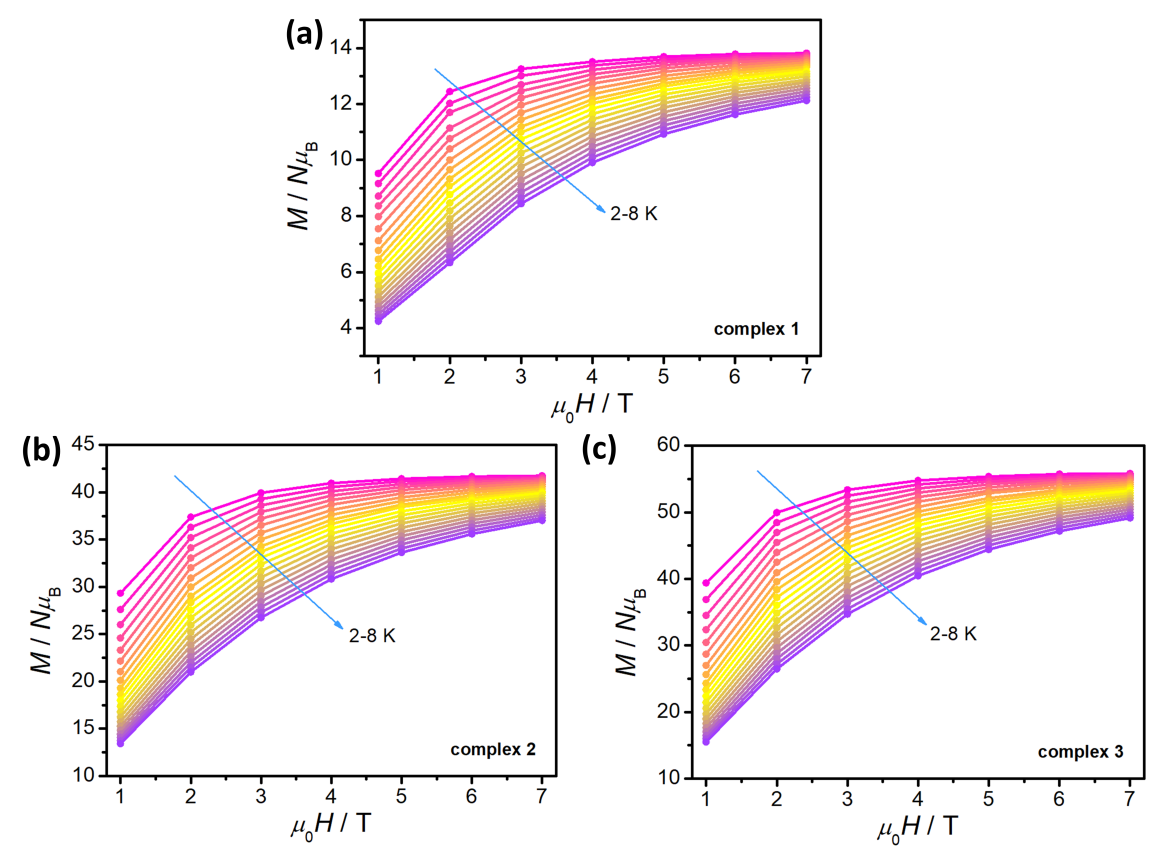


**Supplementary Figure 6.** Variable-field magnetization data at different temperatures for **1**-**3** (a-c).


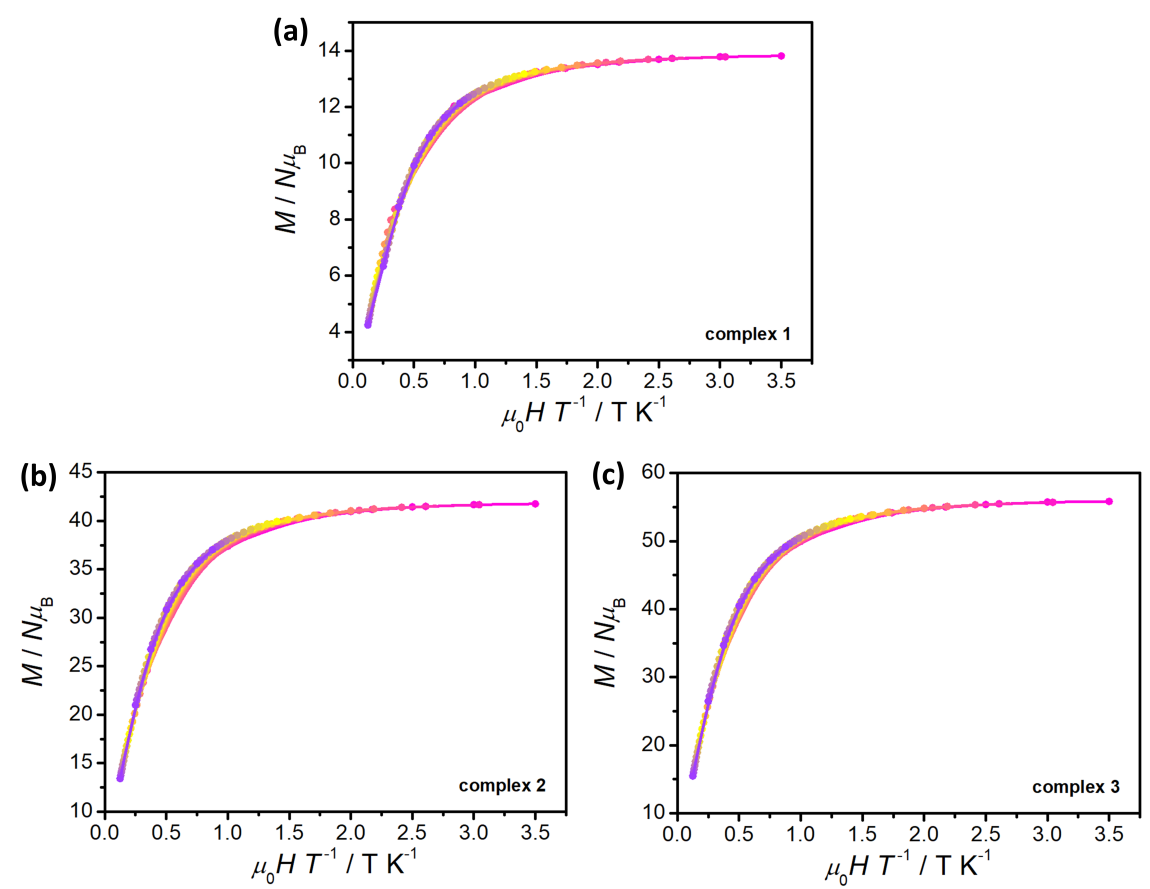


**Supplementary Figure 7.** The plot of *M* *vs*. *HT*^-1^ at different temperatures for **1**-**3** (a-c).


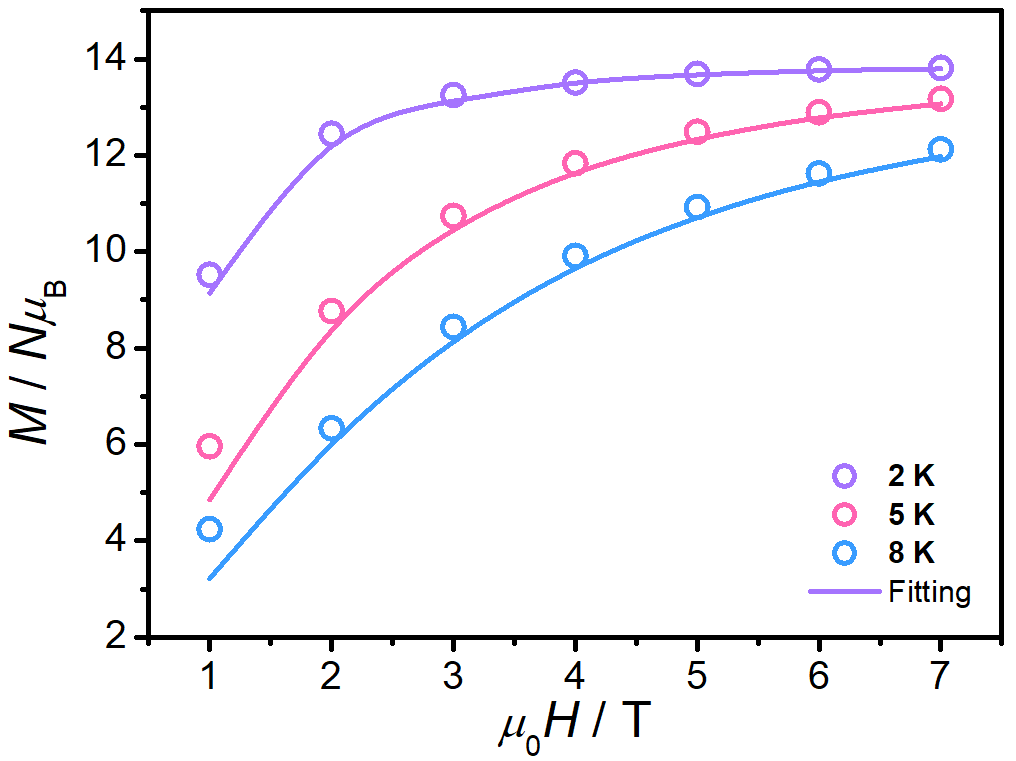


**Supplementary Figure 8.** Variable-field magnetization data for **1**. The solid lines are simulation from the fitted parameters using PHI program. Data were collected from 0-7 T in steady fields.
