# Supplementary material for: Three Gd-based magnetic refrigerant materials with high magnetic entropy: From di-nuclearity to hexa-nuclearity to octa-nuclearity
Source: Front Chem. 2022 Sep 29;10:963203. doi: 10.3389/fchem.2022.963203 (PMC9559567; doi:10.3389/fchem.2022.963203)

checkCIF/PLATON report

You have not supplied any structure factors. As a result the full set of tests cannot be run.

THIS REPORT IS FOR GUIDANCE ONLY. IF USED AS PART OF A REVIEW PROCEDURE FOR PUBLICATION, IT SHOULD NOT REPLACE THE EXPERTISE OF AN EXPERIENCED CRYSTALLOGRAPHIC REFEREE.

No syntax errors found.   CIF dictionary   Interpreting this report

Datablock: Gd8

```
Bond precision:   C-C = 0.0097 A           Wavelength=0.71073

Cell:          a=17.84766(16)     b=18.2321(2)     c=28.0616(3)
               alpha=73.0978(10)  beta=77.7530(8)  gamma=61.2890(11)
Temperature:   120 K
```

	Calculated	Reported
Volume	7634.03(16)	7634.03(16)
Space group	P -1	P -1
Hall group	-P 1	-P 1
Moiety formula	C108 H96 Gd8 N32 O44, O, H2 O [+ solvent]	C108 H96 Gd8 N32 O44, O, H2 O
Sum formula	C108 H98 Gd8 N32 O46 [+ solvent]	C108 H100 Gd8 N32 O46
Mr	3838.19	3840.19
Dx,g cm-3	1.670	1.671
Z	2	2
Mu (mm-1)	3.506	3.506
F000	3700.0	3704.0
F000'	3698.88	
h,k,lmax	25,26,40	25,26,39
Nref	48940	39110
Tmin,Tmax	0.663,0.755	0.760,1.000
Tmin'	0.650	

```
Correction method= # Reported T Limits: Tmin=0.760 Tmax=1.000
AbsCorr = MULTI-SCAN

Data completeness= 0.799           Theta(max)= 31.062
```

R(reflections)= 0.0378(31064)   wR2(reflections)= 0.0946(39110)

S = 1.033   Npar= 1788

The following ALERTS were generated. Each ALERT has the format
test-name_ALERT_alert-type_alert-level.
Click on the hyperlinks for more details of the test.

Alert level B

```
PLAT230_ALERT_2_B Hirshfeld Test Diff for    C19      --C20      .        8.2 s.u.
PLAT241_ALERT_2_B High   'MainMol' Ueq as Compared to Neighbors of        N18 Check
PLAT306_ALERT_2_B Isolated Oxygen Atom (H-atoms Missing ?) .......        O45 Check
PLAT420_ALERT_2_B D-H Bond Without Acceptor  O46      --H46A     .     Please Check
```

Alert level C

```
PLAT213_ALERT_2_C Atom O15              has ADP max/min Ratio .....        3.2 prolat
PLAT213_ALERT_2_C Atom N6               has ADP max/min Ratio .....        3.1 prolat
PLAT213_ALERT_2_C Atom N18              has ADP max/min Ratio .....        3.7 prolat
PLAT215_ALERT_3_C Disordered C65        has ADP max/min Ratio .....        3.6 Note
PLAT220_ALERT_2_C NonSolvent   Resd 1  C   Ueq(max)/Ueq(min) Range         5.1 Ratio
PLAT220_ALERT_2_C NonSolvent   Resd 1  N   Ueq(max)/Ueq(min) Range         5.2 Ratio
PLAT220_ALERT_2_C NonSolvent   Resd 1  O   Ueq(max)/Ueq(min) Range         4.8 Ratio
PLAT222_ALERT_3_C NonSolvent Resd 1  H   Uiso(max)/Uiso(min) Range         4.4 Ratio
PLAT234_ALERT_4_C Large Hirshfeld Difference O15      --C65      .        0.22 Ang.
PLAT234_ALERT_4_C Large Hirshfeld Difference O15      --C65A     .        0.19 Ang.
PLAT234_ALERT_4_C Large Hirshfeld Difference C14      --C15      .        0.16 Ang.
PLAT234_ALERT_4_C Large Hirshfeld Difference C21      --C22      .        0.20 Ang.
PLAT241_ALERT_2_C High   'MainMol' Ueq as Compared to Neighbors of        O15 Check
PLAT241_ALERT_2_C High   'MainMol' Ueq as Compared to Neighbors of        N12 Check
PLAT241_ALERT_2_C High   'MainMol' Ueq as Compared to Neighbors of        N22 Check
PLAT241_ALERT_2_C High   'MainMol' Ueq as Compared to Neighbors of        N26 Check
PLAT241_ALERT_2_C High   'MainMol' Ueq as Compared to Neighbors of        N32 Check
PLAT241_ALERT_2_C High   'MainMol' Ueq as Compared to Neighbors of        C68 Check
PLAT242_ALERT_2_C Low    'MainMol' Ueq as Compared to Neighbors of        C67 Check
PLAT342_ALERT_3_C Low Bond Precision on  C-C Bonds ..............    0.00968 Ang.
```

Alert level G

```
FORMU01_ALERT_1_G  There is a discrepancy between the atom counts in the
            _chemical_formula_sum and _chemical_formula_moiety. This is
            usually due to the moiety formula being in the wrong format.
            Atom count from _chemical_formula_sum:   C108 H100 Gd8 N32 O46
            Atom count from _chemical_formula_moiety:C108 H98 Gd8 N32 O46
FORMU01_ALERT_2_G  There is a discrepancy between the atom counts in the
            _chemical_formula_sum and the formula from the _atom_site* data.
            Atom count from _chemical_formula_sum:C108 H100 Gd8 N32 O46
            Atom count from the _atom_site data:  C108 H98 Gd8 N32 O46
CELLZ01_ALERT_1_G Difference between formula and atom_site contents detected.
CELLZ01_ALERT_1_G WARNING: H atoms missing from atom site list. Is this intentional?
           From the CIF: _cell_formula_units_Z    2
           From the CIF: _chemical_formula_sum  C108 H100 Gd8 N32 O46
           TEST: Compare cell contents of formula and atom_site data
```

```
          atom    Z*formula  cif sites diff
          C        216.00    216.00    0.00
          H        200.00    196.00    4.00
          Gd        16.00     16.00    0.00
          N         64.00     64.00    0.00
          O         92.00     92.00    0.00
PLAT002_ALERT_2_G Number of Distance or Angle Restraints on AtSite         18 Note
PLAT007_ALERT_5_G Number of Unrefined Donor-H Atoms ..............         18 Report
PLAT041_ALERT_1_G Calc. and Reported SumFormula    Strings  Differ     Please Check
PLAT083_ALERT_2_G SHELXL Second Parameter in WGHT  Unusually Large      25.23 Why ?
PLAT176_ALERT_4_G The CIF-Embedded .res File Contains SADI Records          6 Report
PLAT232_ALERT_2_G Hirshfeld Test Diff (M-X)  Gd5      --N27      .        6.5 s.u.
PLAT232_ALERT_2_G Hirshfeld Test Diff (M-X)  Gd6      --N13      .        6.5 s.u.
PLAT232_ALERT_2_G Hirshfeld Test Diff (M-X)  Gd8      --O33      .        5.7 s.u.
PLAT232_ALERT_2_G Hirshfeld Test Diff (M-X)  Gd8      --O43      .        5.3 s.u.
PLAT300_ALERT_4_G Atom Site Occupancy of C65        Constrained at        0.5 Check
PLAT300_ALERT_4_G Atom Site Occupancy of C65A       Constrained at        0.5 Check
PLAT300_ALERT_4_G Atom Site Occupancy of H65A       Constrained at        0.5 Check
PLAT300_ALERT_4_G Atom Site Occupancy of H65B       Constrained at        0.5 Check
PLAT300_ALERT_4_G Atom Site Occupancy of H65C       Constrained at        0.5 Check
PLAT300_ALERT_4_G Atom Site Occupancy of H65D       Constrained at        0.5 Check
PLAT300_ALERT_4_G Atom Site Occupancy of H65E       Constrained at        0.5 Check
PLAT300_ALERT_4_G Atom Site Occupancy of H65F       Constrained at        0.5 Check
PLAT301_ALERT_3_G Main Residue  Disorder .............(Resd  1  )         1% Note
PLAT412_ALERT_2_G Short Intra XH3 .. XHn     H62      ..H65E     .       1.56 Ang.
                                                      x,y,z  =      1_555 Check
PLAT606_ALERT_4_G Solvent Accessible VOID(S) in Structure ........          ! Info
PLAT860_ALERT_3_G Number of Least-Squares Restraints .............          6 Note
PLAT941_ALERT_3_G Average HKL Measurement Multiplicity ..........         3.2 Low
```

```
   0 ALERT level A = Most likely a serious problem - resolve or explain
   4 ALERT level B = A potentially serious problem, consider carefully
  20 ALERT level C = Check. Ensure it is not caused by an omission or oversight
  26 ALERT level G = General information/check it is not something unexpected

   4 ALERT type 1 CIF construction/syntax error, inconsistent or missing data
  25 ALERT type 2 Indicator that the structure model may be wrong or deficient
   6 ALERT type 3 Indicator that the structure quality may be low
  14 ALERT type 4 Improvement, methodology, query or suggestion
   1 ALERT type 5 Informative message, check
```

It is advisable to attempt to resolve as many as possible of the alerts in all categories. Often the minor alerts point to easily fixed oversights, errors and omissions in your CIF or refinement strategy, so attention to these fine details can be worthwhile. In order to resolve some of the more serious problems it may be necessary to carry out additional measurements or structure refinements. However, the purpose of your study may justify the reported deviations and the more serious of these should normally be commented upon in the discussion or experimental section of a paper or in the "special_details" fields of the CIF. checkCIF was carefully designed to identify outliers and unusual parameters, but every test has its limitations and alerts that are not important in a particular case may appear. Conversely, the absence of alerts does not guarantee there are no aspects of the results needing attention. It is up to the individual to critically assess their own results and, if necessary, seek expert advice.

Publication of your CIF in IUCr journals

A basic structural check has been run on your CIF. These basic checks will be run on all CIFs submitted for publication in IUCr journals (*Acta Crystallographica*, *Journal of Applied Crystallography*, *Journal of Synchrotron Radiation*); however, if you intend to submit to *Acta Crystallographica Section C* or *E* or *IUCrData*, you should make sure that full publication checks are run on the final version of your CIF prior to submission.

Publication of your CIF in other journals

Please refer to the *Notes for Authors* of the relevant journal for any special instructions relating to CIF submission.

PLATON version of 19/02/2022; check.def file version of 19/02/2022

Datablock Gd8 - ellipsoid plot

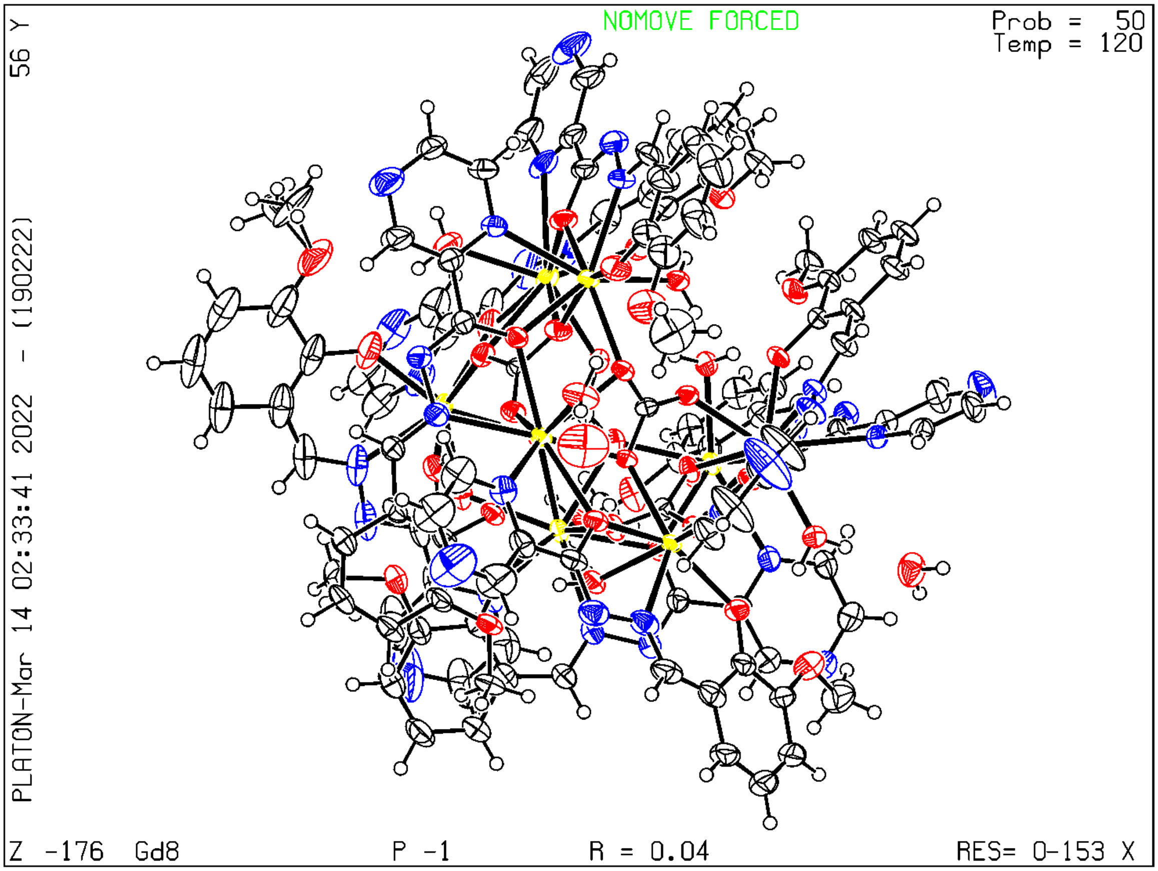

Supplement: Supplementary file 3 [file DataSheet3.PDF]
