# Supplementary material for: Three Gd-based magnetic refrigerant materials with high magnetic entropy: From di-nuclearity to hexa-nuclearity to octa-nuclearity
Source: Front Chem. 2022 Sep 29;10:963203. doi: 10.3389/fchem.2022.963203 (PMC9559567; doi:10.3389/fchem.2022.963203)

checkCIF/PLATON report

You have not supplied any structure factors. As a result the full set of tests cannot be run.

THIS REPORT IS FOR GUIDANCE ONLY. IF USED AS PART OF A REVIEW PROCEDURE FOR PUBLICATION, IT SHOULD NOT REPLACE THE EXPERTISE OF AN EXPERIENCED CRYSTALLOGRAPHIC REFEREE.

No syntax errors found. CIF dictionary Interpreting this report

Datablock: Gd2

Bond precision: C-C = 0.0106 A Wavelength=0.71073

Cell:	a=8.8137(8)	b=9.4123(9)	c=13.3026(12)
	alpha=95.912(3)	beta=109.101(3)	gamma=107.900(3)
Temperature:	120 K		

	Calculated	Reported
Volume	966.55(16)	966.55(16)
Space group	P -1	P -1
Hall group	-P 1	-P 1
Moiety formula	C26 H24 Gd2 N10 O14, 2(C2 H3 N), 2(O)	C26 H24 Gd2 N10 O14, 2(O), 2(C2 H3 N)
Sum formula	C30 H30 Gd2 N12 O16	C30 H30 Gd2 N12 O16
Mr	1129.16	1127.17
Dx,g cm-3	1.940	1.936
Z	1	1
Mu (mm-1)	3.489	3.487
F000	550.0	550.7
F000'	549.90	
h,k,lmax	11,11,16	11,11,16
Nref	3982	3895
Tmin,Tmax	0.665,0.757	0.523,0.745
Tmin'	0.652	

Correction method= # Reported T Limits: Tmin=0.523 Tmax=0.745
AbsCorr = MULTI-SCAN

Data completeness= 0.978 Theta(max)= 26.430

R(reflections)= 0.0379(3622) wR2(reflections)= 0.1001(3895)

S = 1.038 Npar= 274

The following ALERTS were generated. Each ALERT has the format
test-name_ALERT_alert-type_alert-level.
Click on the hyperlinks for more details of the test.

Alert level B

```
PLAT306_ALERT_2_B Isolated Oxygen Atom (H-atoms Missing ?) .......          O8 Check
PLAT971_ALERT_2_B Check Calcd Resid. Dens.  1.03Ang From O8               2.60 eA-3
```

Alert level C

```
CHEMW01_ALERT_1_C  The difference between the given and expected weight for
            compound is greater 1 mass unit. Check that all hydrogen
            atoms have been taken into account.
PLAT043_ALERT_1_C Calculated and Reported Mol. Weight Differ by ..       1.99 Check
PLAT094_ALERT_2_C Ratio of Maximum / Minimum Residual Density ....       2.14 Report
PLAT342_ALERT_3_C Low Bond Precision on  C-C Bonds ...............    0.01064 Ang.
PLAT911_ALERT_3_C Missing FCF Refl Between Thmin & STh/L=    0.600         61 Report
PLAT927_ALERT_1_C Reported and Calculated  wR2 Differ by .........    -0.0011 Check
PLAT971_ALERT_2_C Check Calcd Resid. Dens.  0.23Ang From Gd1             1.77 eA-3
PLAT971_ALERT_2_C Check Calcd Resid. Dens.  1.21Ang From N4              1.51 eA-3
PLAT975_ALERT_2_C Check Calcd Resid. Dens.  0.89Ang From N2      .       0.61 eA-3
PLAT975_ALERT_2_C Check Calcd Resid. Dens.  0.88Ang From O7      .       0.54 eA-3
PLAT975_ALERT_2_C Check Calcd Resid. Dens.  0.76Ang From O4      .       0.53 eA-3
PLAT976_ALERT_2_C Check Calcd Resid. Dens.  0.59Ang From O8      .      -0.86 eA-3
PLAT976_ALERT_2_C Check Calcd Resid. Dens.  0.47Ang From O8      .      -0.80 eA-3
PLAT976_ALERT_2_C Check Calcd Resid. Dens.  0.93Ang From O7      .      -0.66 eA-3
PLAT976_ALERT_2_C Check Calcd Resid. Dens.  1.00Ang From O7      .      -0.63 eA-3
PLAT977_ALERT_2_C Check Negative Difference Density on H7A       .      -0.54 eA-3
PLAT977_ALERT_2_C Check Negative Difference Density on H7B       .      -0.50 eA-3
```

Alert level G

```
PLAT042_ALERT_1_G Calc. and Reported Moiety Formula Strings Differ     Please Check
PLAT068_ALERT_1_G Reported F000 Differs from Calcd (or Missing)...     Please Check
PLAT073_ALERT_1_G H-atoms ref, but _hydrogen_treatment Reported as     constr Check
PLAT083_ALERT_2_G SHELXL Second Parameter in WGHT  Unusually Large      14.44 Why ?
PLAT154_ALERT_1_G The s.u.'s on the Cell Angles are Equal ..(Note)      0.003 Degree
PLAT769_ALERT_4_G CIF Embedded explicitly supplied scattering data     Please Note
PLAT790_ALERT_4_G Centre of Gravity not Within Unit Cell: Resd.  #          2 Note
              C2 H3 N
PLAT790_ALERT_4_G Centre of Gravity not Within Unit Cell: Resd.  #          3 Note
              O
PLAT910_ALERT_3_G Missing # of FCF Reflection(s) Below Theta(Min).          2 Note
PLAT912_ALERT_4_G Missing # of FCF Reflections Above STh/L=  0.600         25 Note
PLAT913_ALERT_3_G Missing # of Very Strong Reflections in FCF ....          3 Note
PLAT960_ALERT_3_G Number of Intensities with I  <   - 2*sig(I) ...          3 Check
PLAT978_ALERT_2_G Number C-C Bonds with Positive Residual Density.          0 Info
PLAT982_ALERT_1_G The Gd-f'=    0.1042 Deviates from  IT-value =      -0.1653 Check
PLAT983_ALERT_1_G The Gd-f"=    4.2731 Deviates from  IT-Value =       3.9035 Check
```

0 **ALERT level A** = Most likely a serious problem - resolve or explain
2 **ALERT level B** = A potentially serious problem, consider carefully
17 **ALERT level C** = Check. Ensure it is not caused by an omission or oversight

```
15 ALERT level G = General information/check it is not something unexpected

 9 ALERT type 1 CIF construction/syntax error, inconsistent or missing data
16 ALERT type 2 Indicator that the structure model may be wrong or deficient
 5 ALERT type 3 Indicator that the structure quality may be low
 4 ALERT type 4 Improvement, methodology, query or suggestion
 0 ALERT type 5 Informative message, check
```

It is advisable to attempt to resolve as many as possible of the alerts in all categories. Often the minor alerts point to easily fixed oversights, errors and omissions in your CIF or refinement strategy, so attention to these fine details can be worthwhile. In order to resolve some of the more serious problems it may be necessary to carry out additional measurements or structure refinements. However, the purpose of your study may justify the reported deviations and the more serious of these should normally be commented upon in the discussion or experimental section of a paper or in the "special_details" fields of the CIF. checkCIF was carefully designed to identify outliers and unusual parameters, but every test has its limitations and alerts that are not important in a particular case may appear. Conversely, the absence of alerts does not guarantee there are no aspects of the results needing attention. It is up to the individual to critically assess their own results and, if necessary, seek expert advice.

Publication of your CIF in IUCr journals

A basic structural check has been run on your CIF. These basic checks will be run on all CIFs submitted for publication in IUCr journals (*Acta Crystallographica*, *Journal of Applied Crystallography*, *Journal of Synchrotron Radiation*); however, if you intend to submit to *Acta Crystallographica Section C* or *E* or *IUCrData*, you should make sure that full publication checks are run on the final version of your CIF prior to submission.

Publication of your CIF in other journals

Please refer to the *Notes for Authors* of the relevant journal for any special instructions relating to CIF submission.

PLATON version of 19/02/2022; check.def file version of 19/02/2022

Datablock Gd2 - ellipsoid plot

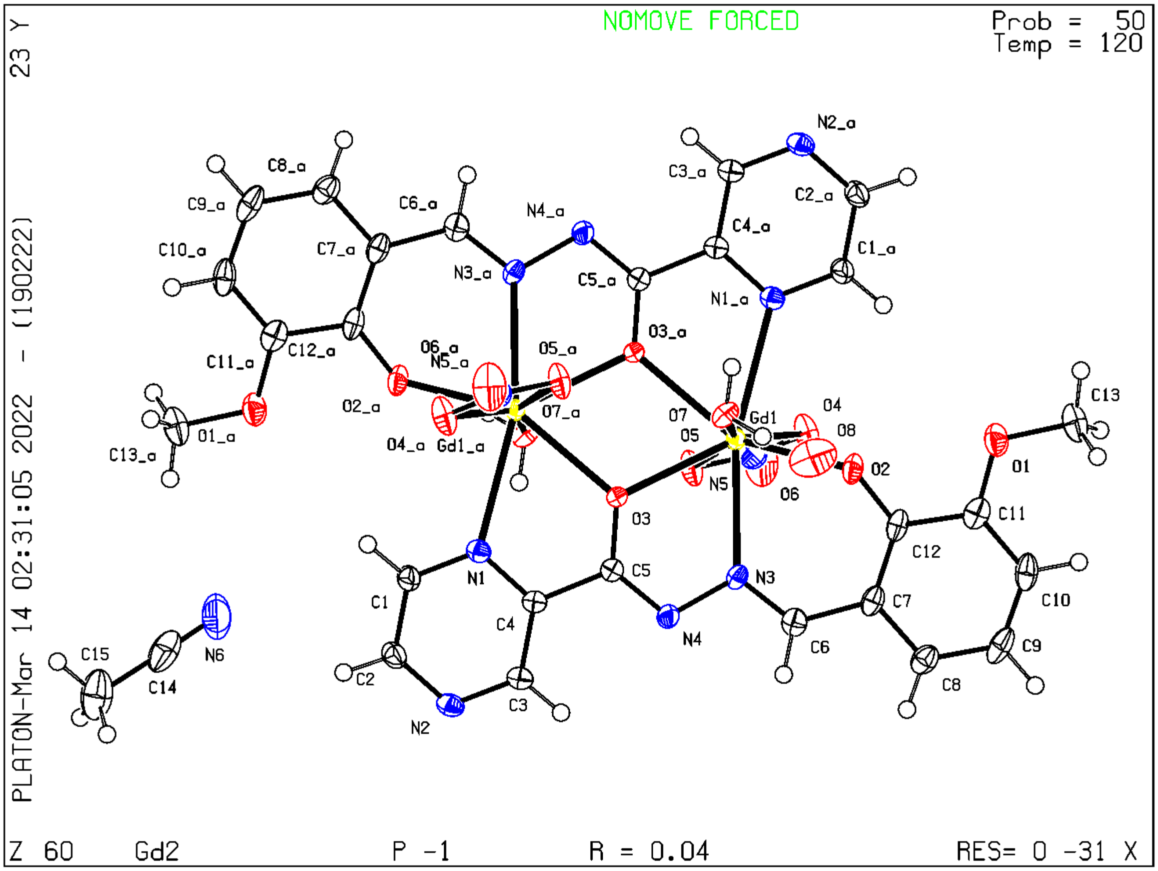

Supplement: Supplementary file 4 [file DataSheet1.PDF]
